# A Decade after the Outbreak of *Xylella fastidiosa* subsp. *pauca* in Apulia (Southern Italy): Methodical Literature Analysis of Research Strategies

**DOI:** 10.3390/plants13111433

**Published:** 2024-05-22

**Authors:** Francesca Serio, Giovanni Imbriani, Chiara Roberta Girelli, Pier Paolo Miglietta, Marco Scortichini, Francesco Paolo Fanizzi

**Affiliations:** 1 Department of Biological and Environmental Sciences and Technology, University of Salento, 73100 Lecce, Italy; francesca.serio@unisalento.it (F.S.); giovanni.imbriani@unisalento.it (G.I.); chiara.girelli@unisalento.it (C.R.G.); pierpaolo.miglietta@unisalento.it (P.P.M.); 2 Council for Agricultural Research and Economics (CREA)-Research Centre for Olive, Fruit and Citrus Crops, Via di Fioranello, 52, 00134 Roma, Italy; marco.scortichini@crea.gov.it

**Keywords:** *Xylella fastidiosa*, Apulia region, One Health, environment, emergency

## Abstract

In 2013, an outbreak of *Xylella fastidiosa* (Xf) was identified for the first time in Europe, in the extreme south of Italy (Apulia, Salento territory). The locally identified subspecies *pauca* turned out to be lethal for olive trees, starting an unprecedented phytosanitary emergency for one of the most iconic cultivations of the Mediterranean area. Xf *pauca* (Xfp) is responsible for a severe disease, the olive quick decline syndrome (OQDS), spreading epidemically and with dramatic impact on the agriculture, the landscape, the tourism and the cultural heritage of this region. The bacterium, transmitted by insects that feed on xylem sap, causes rapid wilting in olive trees due to biofilm formation, which obstructs the plant xylematic vessels. The aim of this review is to perform a thorough analysis that offers a general overview of the published work, from 2013 to December 2023, related to the Xfp outbreak in Apulia. This latter hereto has killed millions of olive trees and left a ghostly landscape with more than 8000 square kilometers of infected territory, that is 40% of the region. The majority of the research efforts made to date to combat Xfp in olive plants are listed in the present review, starting with the early attempts to identify the bacterium, the investigations to pinpoint and possibly control the vector, the assessment of specific diagnostic techniques and the pioneered therapeutic approaches. Interestingly, according to the general set criteria for the preliminary examination of the accessible scientific literature related to the Xfp outbreak on Apulian olive trees, fewer than 300 papers can be found over the last decade. Most of them essentially emphasize the importance of developing diagnostic tools that can identify the disease early, even when infected plants are still asymptomatic, in order to reduce the risk of infection for the surrounding plants. On the other hand, in the published work, the diagnostic focus (57%) overwhelmingly encompasses all other possible investigation goals such as vectors, impacts and possible treatments. Notably, between 2013 and 2023, only 6.3% of the literature reports addressing the topic of Xfp in Apulia were concerned with the application of specific treatments against the bacterium. Among them, those reporting field trials on infected plants, including simple pruning indications, were further limited (6%).

## 1. Introduction

The four major international organizations related to agriculture and environment (i.e., WHO—World Health Organization, FAO—Food and Agriculture Organization, UNEP—UN Environment Programme, and WOAH—World Organisation for Animal Health, have launched the “One Health” 2022–2026 action plan [1] to preserve the health of people, animals, plants and the environment. This is an effort to provide a coordinated response to the new, significant problems that health policies will face in the years to come. What epidemiological studies have long shown, namely the strong relationship between human health, the quality of the environment and animal and plant life, has surfaced most clearly in the context of the COVID-19 pandemic and the climate crisis.

The “One Health” approach acknowledges that the health of people, domestic and wild animals, plants and the ecosystem as a whole are interconnected and tightly related. This balance is particularly demonstrated by the coexistence of insects today, despite the fact that once upon a time they were unknown. One might consider the so-called “alien diseases” spread by invasive species, which, as a result of climate change, are now present in places they previously were not. Invasive species are true phytosanitary crises that are collapsing the agricultural and food production industries. They have severe economic ramifications for many farms and have a significant impact on the management of the areas where the production activities are reliant. They cause economic damage that is ten-times greater than that caused by natural disasters each year, and their number is only going to grow, due to trade globalization and an average global temperature that is warming up, which fosters their invasiveness. With the multiplicity and complexity of ecological issues linked to climate change putting us up against an unprecedented challenge, it is imperative that these issues be evaluated and managed in accordance with the principles of economic and environmental sustainability. It has been almost ten years since the Apulia region in Southern Italy, specifically the province of Lecce, first had to deal with an epidemic that struck this region unprepared and affected the olive tree (*Olea europaea*) [2,3]. Thus, in the early stages of this outbreak, there was a lack of general consensus on the causal agent of the disease. Hence, scientific investigation was necessary and preliminary analysis was asked from the European Commission to EFSA (European Food Safety Authority) with the aim to provide urgent scientific and technical assistance on the plant pathogenic bacterium in this case [4]. After publication of the Scientific Opinion by EFSA’s PLH Panel, which assessed the risk to plant health posed by Xf in the EU territory and evaluated risk reduction options [5], in 2015, the EFSA received a request for an urgent response to scientific and technical information, provided by an Italian non-governmental organization, that claimed that *Xylella fastidiosa* (Xf) was not the cause of the olive tree decline in Lecce Province in Southern Italy. The EFSA Panel on Plant Health subsequently assessed the risk posed by Xf as a pest and examined potential risk mitigation strategies [5]. According to EFSA, Xf has a very wide host range, which includes many common cultivated and wild plants [4]. Insects that feed on xylem fluid are thought to be potential vectors throughout Europe.

In the note by Elbeaino et al. [6] the detection of Xf in adults of species in the Auchenorrhyncha (sharp-shooter leafhoppers and froghoppers or spittlebugs) was reported. In particular, Xf presence was found in *Philaenus spumarius* (Ps) and, for the first time, in *Neophilaenus campestris* (Nc) and *Euscelis lineolatus* (El) (which, unlike the others, is a phloem feeder), indicating the potential vectoring roles of these insects for the spread of the bacterium in the Apulia region. Among them, one clearly recognized vector in Apulia is the polyphagous spittlebug Ps (Hemiptera: Aphrophoridae), which is ubiquitous throughout the entire risk assessment area [7,8,9]. This convincing evidence led to officially recognize the agent of the outbreak as a threat to olive trees in Apulia.

A state of emergency was declared, which was unprecedented not only in Europe but also worldwide [10]. Some of the factors that contributed to this development included the abundance of olive trees in Apulia (ca 65 million, 11 of which are in the Salento peninsula), their size, the widespread presence of the vector *Ps* and the temperate climate, with mild winters, especially in Salento [11]. The bacteria reproduce and live inside the xylem channels of the host plant. They are spread by insects known as vectors, which can travel to a variety of wild and cultivated plants. By suckling sap from diseased plants, the vectors pick up the bacterium and spread it to healthy plants [12]. Diseases on a wide variety of plant hosts are caused by the xylem-limited bacteria Xf [13]. The EFSA’s database of Xf host plants, which originally accounted for 359 plant species (including hybrids) [4], now includes 423 recognized host plants, 194 genera and 68 families [14]. If all plant species, genera and families are taken into account, the figures increase to 679 plant species, 304 genera and 88 families [14]. Italy, specifically the southern portion of the Apulia Region, has been reported as the gateway through which *Xylella* made its entry into Europe. It then passed, with the various subspecies, to the neighboring territories such as the French, Spanish and Portuguese ones [15]. Italy’s host plant diversity includes many common plants, both cultivated and wild. On 22 July 2015, a disease brought on by Xf was first detected in Corsica on *Polygala myrtifolia*, a widely grown and attractive ornamental shrub. Since then, Xf has been found on over twenty different plant species (e.g., *Polygala myrtifolia*, *Laurus nobilis*, *Myrtus communis*, *Myoporum insulare* and *Dodonaea viscosa* ‘Purpurea’) in Corsica and along France’s south-eastern Mediterranean coast [13].

The aim of this paper was to create a thorough analysis of the literature reports that offers a general overview of the specific research field focusing on the Xfp outbreak in Apulia from 2013 to the present. This work will therefore include a methodical analysis of the considered research strategies including the following: the early and further attempts to identify the bacterium; the study to recognize and control the vector responsible for its transportation; the geographical methods used to track disease progression; the development of new diagnostic techniques; the various therapeutic approaches used; the impacts on the environment, man and society and the olive germplasm susceptibility to Xfp. The analysis results should give an overall view of the performed scientific efforts including their distribution among the multitargeted aspects needed to understand and possibly control the disease. The review of the scientific literature and the analysis of data over the last decade could therefore provide useful information for the development of surveillance programs and for suggesting management and control strategies to risk managers, in addition to early detection. This paper also aims at emphasizing the importance of a holistic approach (One Health) to address the crisis of Xfp, combining phytosanitary surveillance, scientific research and cooperation among stakeholders.

## 2. Results and Discussion

**Figure 1:**
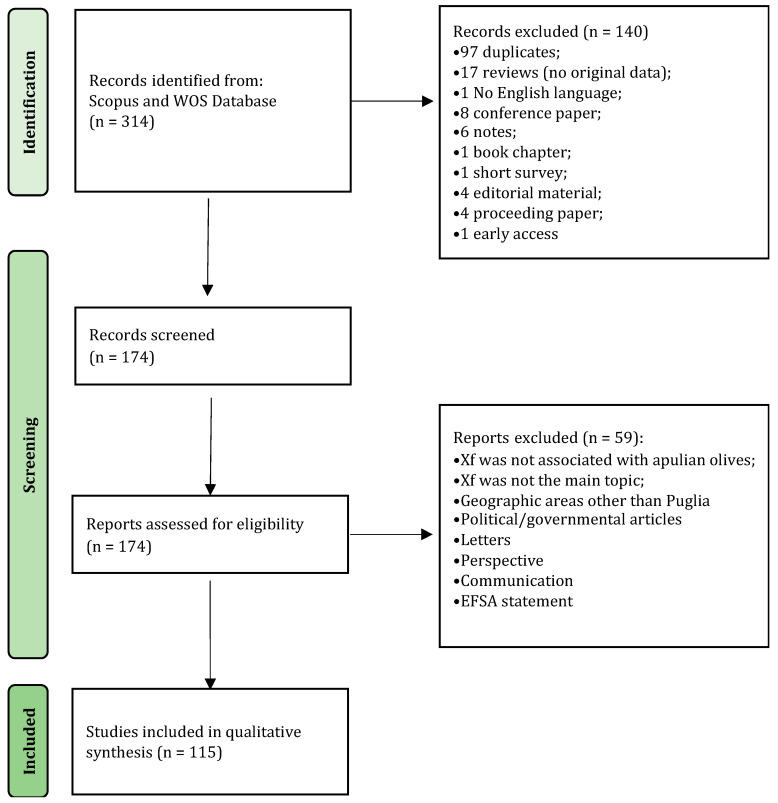
Flowchart of different methodology phases related to the studies included in this review, according to the PRISMA 2020 statement [31].

Details on the selection process are reported in the flowchart (Figure 1). The search in the electronic literature returned 150 records from the Scopus database and 164 from the WOS database. After the process of duplicate removal, 174 references were selected from a preliminary examination of the accessible scientific literature and 115 of them satisfied the inclusion criteria based on the title, abstract and full text screening. These were chosen for the analysis performed in this review and will be briefly summarized and further discussed below, according to the research strategies mentioned above. It should be noted that the present work essentially focuses on the literature analysis of the research strategies used in approaching the Xfp outbreak in the Apulia region, which has been already extensively reviewed in other general aspects [16,17,18,19,20,21,22,23,24,25,26,27,28,29,30].

### 2.1. Xylella fastidiosa, the Bacterium

It was October 2013 when we began to witness the phenomenon of desiccation of plants, secular olive trees of 60–70 years or older, in the first olive groves in Lecce [32], in a large part of the Salento peninsula (cities of Gallipoli, Taviano, Alliste, Felline, etc., on the Gulf of Taranto) at the south-eastern tip of Apulia [33]. It was the first outbreak of Xf under field conditions in the European Union, denoted CoDiRO (abbreviation from the Italian name “Complesso del Desiccamento Rapido dell’Olivo”), due to the presence of a quarantine bacterium, Xf, “responsible” for the syndrome, which affected, and continues to affect, mostly mature plants [2,34]. From 1981 to 2017, Xf was a quarantine pathogen included in the EPPO A1 [35] List of pests recommended for regulation as quarantine pests, but following its introduction in the EPPO region, it was transferred to the EPPO A2 List in 2017 [36]. The presence, in the EPPO region, of different populations and subspecies of the bacterium may allow the appearance of recombining strains, resulting from crosses between the different subspecies. Xf is a gram-negative bacterium [37] belonging to the *Xanthomonadaceae* family, the causative agent of numerous diseases affecting important tree crops, both herbaceous and ornamental [12]; it invades the xylem of a wide range of hosts, from which it is acquired by xylem-feeding insect vectors and transferred to other plants.

In the work by Cariddi et al. [38], it was attempted to isolate the Xf strain in pure culture from symptomatic, naturally infected olive and oleander plants as well as a periwinkle seedling that had been exposed to and infected by Xf-positive spittlebugs. Sequenced PCR products produced from infected periwinkle and oleander colonies were 100% similar to one another and to equivalent sequences obtained from infected olive trees and they shared 97–99% sequence identity with known Xf strains from databases. These sequences formed a distinctive cluster on a branch that included isolates of the Xf subspecies *pauca* (Xfp). As a result, the Apulian Xf isolate was determined to be a strain of the *pauca* subspecies and the name CoDiRO was given to it [38]. Rapid desiccation of the olive tree, whose acronym OQDS derives from the English translation Olive Quick Decline Syndrome, is now the name given to the phenomenon after the crucial etiological role of Xfp was revealed in the following years [39]. According to the Phytosanitary Service of the Apulia Region [40], the disease’s primary symptoms include dieback of twigs, branches or the entire tree; wood browning of the twigs, branches and trunks; and leaf scorch. The partial desiccation of the leaves beginning at the apex and edges, similar to what is seen in the “brusca”, parasitic and non, of the olive tree, is another classic symptom of the *Xylella* disease. Due to the presence of mucilage formed by bacterial colonies and compounds that the plant makes in an effort to fight itself and isolate the pathogen, the woody vessels of the dried branches appear darker. Instead, “brusca” (leaf scorch or marginal leaf burn) is an ailment that affects olives (*Olea europaea*). It is commonly identified by the desiccation and death of tissues at the tip and/or along the edge of the leaf blade, which may be followed by defoliation [39]. Studies were conducted to identify and assess the variety of the Apulian strain of Xf, to comprehend its biology, genetics and phylogeny and ultimately to apply all of this knowledge to more effectively control this disease. Although Xf is thought of as a single species, there are four subspecies that may be distinguished based on DNA differences. These subspecies have different geographic origins and partially different host ranges. The CoDiRO strain is genetically homogeneous, belongs to the subspecies *pauca*, but represents a variety that appears to be identical to a strain from Costa Rica, according to multilocus sequence typing performed on bacterial isolates from axenic cultures [41,42].

The draft genome sequence of the Xf CoDiRO strain, which has a 2,460,000 bp DNA molecule with genes encoding important virulence factors (rpf cluster gene, polygalacturonase-pglA gene), was determined by Giampetruzzi et al. [43] confirming this taxonomic allocation.

In order to analyze the Xf strain from the Apulia region, Mang et al. [44] looked at the 16S rDNA gene, the gene encoding the B subunit polypeptide of the DNA gyrase (gyrB) and the putative protein HL gene [45]. In order to examine the nucleotide diversity of the Apulian strain of Xf and its connections to other Xf species, 16S rDNA, gyrB and HL gene sequence comparisons as well as phylogenetic analyses were carried out. It was possible to distinguish between the subspecies *pauca* and *fastidiosa* of Xf using HL nucleotide sequences. However, the gyrB gene’s nucleotide change helped researchers to distinguish Xfp from the two subspecies multiplex and *fastidiosa*. Based on the phylogenetic analysis of three genes, the Xf strain from the Apulia region was classified as the subspecies *pauca*. Based on multilocus sequence typing, Loconsole et al. [46] and Martelli et al. [47] reported the interception of three novel bacterial sequence types in Italy that cluster with various Xf subspecies, highlighting the danger of introducing more pathogen genetic diversity into Europe. They specifically stated that infected plants in the provinces of Lecce and Brindisi were linked to a single genotype (ST53), which had already been identified in Italy and Costa Rica [41,42]. Additionally, ST53 (CoDiRO strain), the same sequence type of this bacterium in five other plant species sampled from the infected zone, was discovered to be present in all of the new host plant species positive for Xf, including cherry, myrtle-leaf and rosemary. This evidence lends further support to the theory that coffee plants imported from Central America that were infected with Xf were the cause of the epidemic.

Marcelletti and Scortichini [48], in their study, assessed the complete and draft genome of 27 Xf strains and the draft genome of *X. taiwanensis*. They demonstrated the occurrence of a genetic clonal complex of four Xf strains belonging to the subspecies *pauca*, which evolved in Central America. This clonal complex includes the CoDiRO strain, which was linked to the “olive quick decline syndrome” and was discovered in southern Italy. It consisted of two strains isolated from Nerium oleander and one strain derived from *Coffea* sp. This study strongly supports the possibility of the introduction of Xf in southern Italy through coffee plants grown in Central America.

After this research, Cella et al. [49] used BEAST (Bayesian Evolutionary Analysis Sampling Trees) to use phylogenetic and evolutionary analysis to determine the potential origin and route of the introduction of two bacterial strains (*X. fastidiosa* subs. *pauca* and *X. fastidiosa* subsp. *sandyi*). These two bacterial strains likely had two separate pandemic introductions, according to phylogenetic research. The two separate routes of provenience for Xfp from Costa Rica (posterior probability 100%) and Xf subsp. *sandyi* (Xfs) from California (posterior probability 100%) were also disclosed and validated by the phylogeographic study [50]. The current hypotheses for the provenience of Xfp [50] and the geographic origin of X have both been supported and validated by phylogeny. Additionally, Giampetruzzi et al. [50] chose and characterized the full genome sequence of the Xfp strain De Donno (CFBP 8402) among the ST53-cultured isolates obtained from OQDS-affected olive trees. Under experimental circumstances, this bacterium, manually implanted in various cultivars of olives, caused symptoms that were the same as those seen in contaminated olive groves. Furthermore Saponari et al. [51] determined Xf-OQDS relationships via bacterial isolations in axenic culture. Then, in order to test the potential of the cultured bacteria to colonize these specific hosts and mimic the symptoms seen in the field, plantlets of the myrtle-leaf milkwort (*Polygala myrtifolia*), oleander (*Nerium oleander*) and olive (*Olea europaea*) species were mechanically inoculated with the cultured bacteria. The relationship between OQDS, diseases of oleander and myrtle-leaf milkwort and Xfp strain De Donno was experimentally demonstrated for the first time in this study, which also showed that this bacterial strain can cause the same symptoms in hosts as reported with in the field observation. In fact, Xf-free olive plants were subjected to mechanical and graft inoculations that caused wilting, dieback and eventual death of the plants.

Multiple loci VNTR analysis assay (MLVA assay), created by Mazzaglia et al. [52], is a molecular method for identifying various pathogen populations. In particular, this method checks, in silico, the presence of the VNTR loci reported in the literature within the completely edited genome of the “De Donno” strain. The MLVA assay also demonstrated its ability to produce new and deeper data about the genetic diversity of this subspecies, with particular reference to the Italian outbreak in Apulia. The assay distinguished between subspecies or even sequence types (STs) with clarity, but it also identified variations within the same ST to give more specific details on the dynamics and pathways for pathogen dissemination. It is especially helpful for mass screening of infection and for enhancing containment measures due to its effective application even on whole DNAs taken from infected tissues of various host plants.

Furthermore, in the research by D’Attoma et al. [53], the reference strain of the subspecies *fastidiosa*, Xf Temecula1, which was isolated from California grapevines, was used to compare the in vitro performance of Xfp strain De Donno [54,55]. Using transformation protocols, the olive strain’s natural competence ability and suitability for genetic modification were evaluated. This information will be helpful in the development of bacterial mutants that serve as a useful tool for studying the interactions between the strain and its plant hosts as well as the vector. The tests unmistakably showed that the strain De Donno was more aggregative than the strain Temecula1, formed more biofilm and did not exhibit fringe on the agar plates.

Understanding how adaptive features have changed over time requires a close and ongoing observation of this invading pathogen’s genetic variation. Also emphasized was the significance of adopting all practical safety measures to reduce the possibility of upcoming introductions that could lead to an increase in pathogen diversity. In 2018, Ramazzotti et al. [56] used DNA typing techniques that target the entire pathogen genome to study for the first time the occurrence of sub-variants within a set of 14 “ST53” isolates of Xf collected from various areas. The three Xf isolates from Salento (Apulia, Italy), namely Salento-1, Salento-2 and De Donno, whose complete genome sequences were released, share a very recent common ancestor. All tested isolates shared the same genomic fingerprint, supporting the existence of a predominant epidemiological strain in Apulia.

The epidemiological characteristics of OQDS and Xfp in Apulia were mainly unclear until 2019 despite a significant number of studies being conducted to clarify several factors connected to the bacterium, such as its taxonomy, origin and host range. The Apulia Region’s Agenzia Regionale per le attività Irrigue e Forestali (ARIF) inspectors monitored the containment and buffer areas of Taranto and Brindisi provinces between September 2017 and March 2018.

Scala et al. [57] explored the lipid composition of Xfp strain De Donno in pure culture by LC-TOF (liquid chromatography coupled with time-of-flight mass spectrometry) and LC-MS/MS (liquid chromatography–tandem mass spectrometry). They used *Nicotiana tabacum* to test the ability of Xfp to produce specific lipids (e.g., ornithine lipids and oxylipin 7,10-diHOME) in the host. Different lipid compounds were revealed and showed a distribution pattern within the infected plant tissues compared to the uninfected ones.

Scortichini and Cesari [58] analyzed and discussed the official data regarding 220,279 olive trees. In this study, the most prevalent signs of OQDS and Xfp in olive trees were leaf scorch and twig and branch dieback. These symptoms are thought to come before severe crown collapse and plant mortality. In total, 8.06% of the 220,279 olive trees monitored in those regions had leaf scorch, twig diebacks and tiny branch diebacks; some cultivars predominantly planted in the examined areas appeared to have more symptoms than others. In fact, the cultivars “Nociara”, “Cima di Melfi” and “Cellina di Nardò” displayed the highest frequency of decline symptoms and it appears that tree age is connected to the incidence of decline symptoms.

In 2020, Scala et al. [59] explored for the first time the lipidomic profile of olive trees naturally infected by *Xylella fastidiosa*. They examined by untargeted analysis all the lipids that change under the pathogen pressure and differentiated the infected trees from the uninfected ones. The difference in clustering between OQDS-negative and OQDS-positive samples suggests that Xfp presence can influence the differential formation of lipid entities. Although the biology of Xfp infections has been investigated, there is still a lack of knowledge regarding the factors that determine specificity between bacterial genotypes and host plant species, which is especially important now given the spread of Xfp. Firrao et al. [60] scanned the Xf pan-genome to identify the genes that are not coherent with its phylogenetic position within the order Xanthomonadales. The results of the analysis revealed that for a significant part of the gene, the closest homologue was found in bacteria belonging to distantly related taxonomic groups. Although the ability of Xf strains to recombine themselves is well known, the results of the pan-genome analyses stressed the additional relevance of environmental DNA in shaping their genomes, with potential consequences on their phytopathological features. In the affected area of Italy, Sicard et al. [61] examined the genomes of 79 Xf samples taken from sick olive trees (*Olea europaea* cv. “Cellina di Nardò” or “Ogliarola Salentina”). By combining population genomic approaches with evolutionary genomics techniques, they demonstrated that the outbreak in Apulia is the result of a recent single pathogen introduction (in 2008) from Central America and identified a small list of genes that may be crucial in Xf’s environmental adaptation. The main results related to its identification and phenotypic and genotypic characterization are summarized in Table 1.

### 2.2. *Xylella fastidiosa* Vectors

Xf requires an insect vector to spread from an infected plant to a healthy one; in particular, it is spread by hemipterans that feed on xylem fluids, such as cicadellids and aphrophorids (cercopids) [62,63,64]. Since it is a bacterium that lives in the xylem, only xylem fluid-eating insects can act as a vector for Xf. The food canal (precibarium) and pumping chamber (cibarium) in the foregut of the insect vector are where the bacteria adhere and grow after being ingested by the insects [65]. There is no trans-stadial or trans-ovarial passage of the bacterium. Only a small number of bacterial cells are required for transmission and there is no latent period. The vector in Italy was unknown, despite the fact that numerous species of spittlebugs and sharpshooters that feed on xylem fluids are known to spread the bacteria globally. But the September 2013 finding of Xf in southern Italy sparked a rapid search for insect vectors of the bacterium. Saponari et al. [7] conducted the first transmission experiment using field-collected meadow spittlebugs from Gallipoli (Salento, Apulia region) caged in groups of 8–10 per plant on five periwinkle (Catharanthus roseus) plants and seven olive (*Olea europaea*) plants for an inoculation. The authors reported the first findings of field-collected *Olea europaea* L. (Ps) positive by PCR polymerase chain reaction (PCR) experiments on adult Ps that revealed that two out of every five periwinkles tested positive for Xf, but transmission to olives was not achieved. The well-known color polymorphism of the genus *Philaenus* makes it possible to distinguish between morphs and involves unskilled practitioners in species identification. The study by Lahbib et al. [66] suggests that Ps has twenty-three morphs, followed by eight morphs of *P. signatus*, seven of *P. tesselatus*, six of *P. italosignus*, *P. maghresignus* and *P. tarifa*, two of *P. loukasi* and one of *P. arslani*. Ps morphs are also known as Spitterbugs. This is due to the chrysalis being coated in a saliva-like fluid that is secreted by glands located on the abdomen. This saliva reacts with the air to create a frothy mass that helps the nymph stay cool and hide from predators. Even though these initial findings should be regarded as being of the utmost significance, the authors themselves suggested that additional research is necessary, particularly in light of the fact that Xf was not transmitted from Ps to olives and that the candidate vector survey was only conducted during the coldest three months of the year, when the majority of the species’ biological cycle should be regarded as having been completed. Cornara et al. [8] conducted a number of studies and field sampling to learn more about the potential contribution of the prospective vectors to the Xf epidemiology in the area. Ps was the only species that consistently tested positive for the presence of Xf during the survey, and it was the most prevalent species on non-olive vegetation in olive orchards, as well as on olive leaves. However, tests showed that Ps spread Xf from infected to uninfected olive plants after the first Xf-infective Ps was discovered from an olive canopy. Additionally, Ps picked up Xf from a variety of host plant species, with olives, polygala and acacia showing the highest rates of acquisition.

Unfortunately, this vector species is extremely widespread and polyphagous; the nymphs can consume a wide variety of herbaceous plants and the adults disseminate to an even wider variety of plant species, including a large number of trees and shrubs [67,68]. Although other perennial hosts have been found, mostly in non-cultivated places, such as gardens and the natural landscape, the olive is the most common host in the contaminated areas. However, because both Xf and Ps have a wide host range and plant community composition, their role is significant in the epidemiology of Xf-associated diseases. Cornara et al. [9] conducted studies to assess Ps natural infectivity in olive orchards infected by Xf and evaluated the transmission of Xf by Ps to different host plant species and cultivars. Additionally, these data demonstrate that field-collected Ps have high rates of Xf infection because the proportion of Ps infected with Xf ranged from 25% to 71% throughout the entire survey period and the number of Xf cells detected in Ps head ranged from 3.5 × 10 to 4.0 × 10^2^ (CFU equivalents). Xf was specifically detected in all the host plants after insect plant access with the exception of grapevines. In olive orchards, Moussa et al. [69] describe the seasonal abundance of various Auchenorrhyncha species and the temporal dynamics of insect Xf infection. Over the course of two years, adult populations of the species Ps, Nc and El were observed monthly in five olive orchards. The predominant species Ps had the maximum adult abundance in the summer (39.8% of all Auchenorrhyncha caught). Between March and May in both years, adult Ps and *N. campestris* were initially discovered, and every insect tested during these times responded negatively to Xf by PCR. In four olive orchards in coastal and inland regions of Apulia, Bodino et al. [70] studied the structured population phenology, abundance and seasonal movement between crops and wild plant species of both the nymphal and adult stages of various spittlebug species. Ps was the most prevalent species in this study as well; in late spring, Ps adults were numerous on the olive trees and herbaceous cover.

In the summer, they dispersed to wild woody hosts before returning, in the fall, to the olive groves to find oviposition sites in the herbaceous cover. Additional experiments have been conducted over the years to evaluate their seasonal variations and to comprehend their potential impact to the epidemiology of the illnesses on olives. More specifically, adults of the spittlebugs *P. italosignus* (Pi) Drosopolous and Remane, Nc (Fallen) and of the planthopper *Latilica tunetana* (Matsumura) (Issidae) have been tested in transmission experiments to assess their ability to acquire the bacterium from infected olives and to infect different susceptible hosts (olives, almonds, myrtle -leaf milkwort, periwinkle). The involvement of two additional spittlebug species, Nc and *P. italosignus*, as a vector of the Apulian strain of Xfp was examined by Cavalieri et al. [71]. Individual insects were tested in quantitative PCR assays to evaluate acquisition rates, which ranged from 5.6% in Nc to 22.2% in Pi, although no acquisition was noted for *L. tunetana*. In order to grasp the close link that exists between the meadow spittlebug and Xfp, it may be helpful to have a thorough understanding of the Ps feeding habits. Since stylets are inserted into plant tissue during a probe, it is impossible to directly observe insect activity; all published data on Ps feeding behavior up until 2018 have been generated using techniques that only provide snapshots and indirect evidence of the complete process. In their work, Cornara et al. [26] used the Electrical Penetration Graph (EPG) method to characterize Ps in real-time. Ps males were subjected to additional EPG recordings in order to examine any potential sex-related variations. The five different key waveforms that make up Ps’ feeding behavior are route, xylem contact/pre-intake, xylem sap ingestion, resting and disruption during the xylem phase. Females take substantially longer to consume xylem sap than males do, although it takes them less time to start the first probe. The transmission dynamics of Xf by Ps were investigated in a different study by Cornara et al. [72] employing EPG in tests to investigate the link between vector feeding behavior and transmission. The use of EPG has proven to be essential in identifying the behaviors connected to the acquisition and immunization of numerous vector-borne plant diseases over time. When Ps consumed xylem or engaged in activities interspersed with xylem ingestion (resting while xylem was consumed), bacterial cells bound to the insect’s foregut at a low rate (about 15 min). For Ps to transmit Xf, a relatively short period of time is therefore necessary. Based on their eating habits, cicadas, including *Cicada orni* L. (1758) (Hemiptera: Cicadidae), one of the most frequent Hemiptera that live in these agro-ecosystems [73], may also be a potential vector for the bacterium. Cornara et al. [74] discovered that none of the recipient olive plants, whether they were caged with groups of three *C. orni* individuals per plant kept in sleeve cages (55 plants) or placed inside a mesocosm with cicadas free to move among the recipient plants (30 plants), were infected with the bacteria when *C. orni* were collected from infected olive plants to see if they could spread the bacteria to healthy olives. Additionally, only 4 (1.27%) of the 314 field-collected *C. orni* that were tested by qPCR for the bacteria were positive. As a result, the role of the cicada species in the Xf’s natural distribution is either nonexistent or very small. The study by Fierro et al. [75] proposes a lattice model for the pathogen invasion of olive orchards with the goal of determining an appropriate strategy to halt the infection. This strategy is based on managing the vector throughout its entire life cycle to explore the extent to which the infection can be mitigated even in unfavorable conditions.

This study makes it abundantly evident that simple vector management, which attempts to regulate the vector population size (on eggs, juveniles and adults), differs from transmission management, which tries to lessen the possibility that adults will spread events that trigger controls against juveniles. The control of the Xf invasion is particularly problematic because the infected region precedes the symptomatic ones by many kilometers due to the delayed symptoms of infection. Because the eventual diagnosis of Xf infection and the search for it are symptom-dependent, the state of alert is declared too late and too far away from the invasion line to be useful. The spread of Xf illnesses is the result of intricate biotic and abiotic interactions and it is difficult to forecast. However, the rate of dissemination is influenced by a number of variables, including the population size of capable vectors. The presence of a ground cover and the species makeup of that ground cover, which can be more or less beneficial or attractive to spittlebugs, are the key factors controlling the vector population level in olive orchards. Despite a wealth of literature documenting the hosts of Ps nymphs, such as Weaver and King’s list [67] of more than 200 plant species from 1954, there is little information on the preferred hosts of this species in the Mediterranean region. Similar results show that *Neophilaenus* spp. nymphs are linked with gramineous plants [76], although there is no information on the host plant preferences of these latter. In order to identify the plants that, both inside and nearby olive groves, can act as reservoirs of the vectors, Dongiovanni et al. [77] described host plant selection of spittlebug nymphs under field conditions in Apulian olive groves located both inside and outside the Xf-infected area. The findings showed that, in comparison to olive groves in the Bari province (a non-infected area), the rate of plant infestation by spittlebug nymphs in 2016 was much greater in the province of Lecce (an infected area). Asteraceae, Fabaceae and Apiaceae were the botanical groups that included the greatest proportion of plants afflicted with Ps nymphs. The nymphal populations of three species of spittlebugs (Ps, *N. campestris* and *Aphrophora alni*) and the herbaceous cover were estimated by Bodino et al. [78]. The dominating species was Ps. Additionally, they noted that nymphal stages are extremely polyphagous and favor several species, such as Picris, Crepis, Sonchus, Bellis, Cichorium and Medicago, within the Asteraceae Fabaceae plant family. Very little information has been published on the ability of Ps or other Aphrophoridae species to disperse, despite the significance of vector dispersal in the spread of Xf. Adult spittlebugs frequently leap or walk instead of flying, which is why they are thought to be poor flyers [26,67]. Despite being a crucial factor in predicting the spread of the bacterium, little is known about Ps ability to disperse. The movement of Ps adults in an olive grove in Apulia (Southern Italy) was studied by Bodino et al. in 2021 [79]. Ps may only move a few hundred meters at a time throughout the entire year; in the olive grove, the median distance from the release site for a day of dispersal was 26 m. They calculated that, at the time of peak vector abundance on olives, 50% of the spittlebug population persisted within 200 m and 98% within 400 m. The secondary transmission of the bacterium from olive to olive occurs in Apulian olive orchards where adults of Ps disseminate toward olive plants shortly after emerging on ground cover plants [8]. The olive serves as both a source and a reservoir plant for Xf. In Apulia, during the phenological stage of flower emergence in May and June, adults are numerous on olive canopies. After that, Ps disperse toward suitable wild trees and shrubs surrounding the olive orchard when olives are no longer suitable, possibly as a result of changes in plant physiology and chemistry driven by the harsh summer climatic conditions (high temperatures and drought stress) [26,78]. In a study region between 60 and 100 km north-west of the front of the Xf-infected area, Cornara et al. [80] described the abundance of spittlebugs on wild trees and shrubs throughout the year. Spittlebug adult populations peaked in a pine forest and along a lakeshore, the latter of which was dominated by elm trees, between June and September, with peak numbers on elms occurring in July and August. Nc, Ps and Pi Drosopoulos et Remane, three species of spittlebugs, were equally distributed on pine trees, whereas the latter was more prevalent on elms. On elms, a few individuals were gathered throughout the year, but spittlebugs on pine trees were only gathered in the summer. In order to better understand the kinetics of the bacterial persistence, transmission effectiveness and the spread rate of Xf among olive trees in summer and autumn, Bodino et al. [81] conducted two sets of experiments to study the transmission biology of Xf by Ps. Their findings demonstrate that Ps is a capable Xf vector to olives throughout its adult life. The bacteria load in the vector foregut increases during the first 2–3 weeks after acquisition and then stabilizes. Transmission rates may significantly vary throughout the year and under different climatic conditions. The ongoing monitoring and surveillance of its insect vector is essential for the efficient management of this biological intruder. Thus, Bozzo et al.’s research [82] identifies the invasion factors (i.e., landscape and vegetation variables) that affect the quantity and dynamics of this vector and, as a result, the spatial expansion of this bacterium in this Italian region. In order to do this, a spatial pattern clustering analytical approach was taken into consideration. The findings showed that territorial differentiation and geographical variation may vary from zone to zone within the same invaded region, which calls for effective management and monitoring planning. The modulation of the target insect’s foraging behavior and that of its close natural enemies is one example of an innovative technique in pest control. Essential oils (EOs) have been shown to play a role in both direct and indirect plant defenses against herbivores and pathogens [83]. EOs are a significant source of bio-active volatile compounds [84] that are biosynthesized in various plant organs and have the ability to interfere with basic metabolic, biochemical, physiological and behavioral functions of insects [85]. In their study, Ganassi et al. [86] investigated the behavioral reactions of the spittlebug Ps to a variety of essential oils (EOs) and aromatic plants. The chosen EOs caused antennal reactions in Ps males and females, showing that both sexes’ peripheral olfactory systems can detect the volatile compounds present in these EOs. This finding supports the hypothesis that EO volatiles may serve as long-distance cues to Ps adults. In this context, it is also advantageous to identify the exact mixture of volatile organic compounds (VOCs) that a host plant releases in order to direct pest insects away from it and toward other targets (plants, traps). When the grass cover dries up, Ps, the primary vector of Xf, shifts to olive trees. The potential function of chemical signals in controlling this passage is unknown as of yet. In olfactometer bioassays, Cascone et al. [87] described Ps’ behavioral response to two resistant (Leccino, FS-17) and three widely grown susceptible kinds of olive tree (Ogliarola, Frantoio and Rotondella). Ps had different responses for males and females depending on the different olive varieties. Females were drawn to Ogliarola, Rotondella and Frantoio while being repulsed by FS-17. The behavioral observations were supported by the characterization and quantification of the VOCs released by the tested olive varieties, which opened the door to discussion about their potential contribution to the development of integrated protocols for the long-term defense of olive trees against the lethal spread of Xf.

The vector identification, bionomics, infection management and induced disease by the *Xylella* invasion have already been extensively reviewed [18,88]. The main findings related to Xf vectors, related to its identification, characterization, life cycle, control and management strategies, are summarized in Table 2.

### 2.3. Geographical Methods

Models of species dispersal might offer plausible explanations for how bioclimatic factors affect newly developing plant diseases. Xfp ST 53, the cause of olive quick decline syndrome [2], is a new invasive strain that was previously limited to the Americas. Since its discovery in October 2013 near Gallipoli, southern Italy [41], it has spread over 100 km and killed about 4 million olive trees and it is still growing. According to estimates, the bacterium infected 8000 hectares of olive orchards in 2013, the year the disease was initially reported [2,89], but one year later, in December 2014, the known infected area had nearly tripled [90].

In Apulia, a strategy to track disease progression and stop the emergence of fresh, advanced foci has been in effect since November 2013. To determine whether Xf is present, samples of olive trees and other known sensitive host species have been collected. The sampling strategy is based on the delineation of three so-called “demarcated areas”: the infected zone, which is the area containing all known infected trees; the containment zone, which is the 20 km wide northern part of the infected zone where infections are frequently found and specific control operations are required; and the buffer zone, which is the 10 km wide disease-free area immediately outside of the containment zone where strict preventative measures are taken.

The dynamics of the infection of Xf, the bacteria that has had a major impact on landscapes in the Apulia region over the past decade, are briefly summarized in Table 3. A preliminary examination of the pathogen’s possible geographic distribution was conducted by Bosso et al. [91], who identified the eco-geographical factors that would favor the disease occurrence in Italian regions other than Apulia. The research demonstrated that Xf has a high probability (>0.8) of colonizing areas characterized by low altitude (0–150 m a.s.l.), precipitation ranging between 80 and 110 mm in the wettest month, limited to 60 mm in the warmest quarter of the year, and with a mean temperature of 8 °C in the coldest quarter.

Land cover analysis also revealed that Xf might essentially occur in the following: (i) agricultural areas (75.5%) with intensive agriculture, complex cultivation patterns, olive groves, annual crops associated with permanent crops, orchards and vineyards; (ii) forest (12.8%), mostly oak woodland; and (iii) Mediterranean shrub-land (11.8%). Furthermore, in the most significant olive-growing regions of the Mediterranean Basin, climate change appears to set off environmental circumstances that are particularly conducive to the spread of the bacteria [91]. The epidemic’s Apulian-monitoring program collected data from tens of thousands of lab tests looking for the presence of the bacterium together with georeferenced sample information. Bucci [92] used these data as a starting point to demonstrate how Xf spreads by producing new, densely packed clusters of infected plants, with 98% of the diseased trees being separated by less than 100 mt from another infected tree. Since more than three quarters of the newly detected epidemic hotspots resulted farther than 1 km from any previously known infected plants, possible underestimation of long-range spreading of the bacterium or the need to revise the monitoring strategy was also suggested.

Several models were created to analyze and forecast the spread of Xf. White et al. [93] created a spatially explicit simulation model for Xf in order to guide management techniques and provide assistance for anticipating the spread in the early phases of invasion. The spread patterns were predicted by the model both qualitatively and quantitatively. According to the data, it was found that increasing buffer widths reduce the likelihood of infection outside of the control zone. Nevertheless, due to stochastic long-distance leaps brought on by vector dispersal, the spread may not be totally stopped. In 2020, White et al. [94] adopted a Bayesian approach to infer epidemiological parameters by fitting and comparing compartmental epidemiological models to brief snapshots of disease progression seen in numerous field plots. An estimation of the potential for each infected tree to infect about 19 more trees annually as well as a desiccation occurring about 4.3 years after symptoms first appeared were also reported. On the other hand, the symptomless stage was predicted to have low to insignificant infectivity and persist for, on average, 1.2 years.

The major goal of the study by Castrignanò et al. [95] was to define a geostatistical approach of data fusion. They combined visual inspections, plant diagnostic tests and both remote (radiometric) and proximal (geophysical) data to produce probabilistic maps of Xfp infection risk. The drone data-supported method allowed for the identification of potential infection entry points into the field as well as the marking of regions where the risk of infection was higher. The visual evidence supported the findings from the drone data; the withering process propagated almost randomly, even within the same plant, making it very challenging to identify favored paths of disease diffusion. On the other hand, drone surveillance showed that olive trees exhibit high levels of variety, with varying leaf vigor coexisting on the same tree. In the study by Cendoya et al. [96], the influence of climatic variables on the geographic distribution of the pathogen as well as the spatial association between the positive locations in each region were determined using a Bayesian geostatistical model. The majority of positives were found in Lecce’s south-west region, where they coincided with high annual mean temperatures and mean temperatures of the wettest quarter and low values of mean diurnal range, mean temperature of the driest quarter, annual precipitation and precipitation of the driest month. Interestingly, Xf is currently found in regions with a variety of climatic conditions [19]. Although the infection is most prevalent in areas with tropical and subtropical climates, it can also be found in far colder and/or drier areas. Kottelenberg et al. [97] analyzed the hundreds of thousands of records of monitoring data on illness occurrence in the Apulia region to determine the nature of the invasion front and its rate of movement. The obtained estimation’s robustness was also examined with specific simulations. The results indicated a disease front moving at a rate of 10.0 km per year, within a 95% confidence band of 7.5 to 12.5 km per year. According to the fitted model, the spreading of the illness probably began around 2008. Effective management of new foci requires an understanding of the dynamics of the Xf infection. A recent Eco-epidemiological Model (XEM) by Gilioli et al. [98] was proposed to describe the dynamics of infection in Xf outbreaks. The simulation results of the XEM, a spatially explicit mechanistic model for the short-range spread of Xf, revealed that the vector’s abundance is the primary determinant of the pathogen’s rate of spread. Therefore, the important elements for a successful eradication strategy were identified as vector control efficacy and time to detection and intervention. Using an ecological niche model, Bajocco et al. [99] examined how the distribution of Xf-infected olive trees changed between 2015 and 2021 across the Apulia region, with different land uses as a proxy for human pressure index [100]. The road system served as the primary engine of diffusion while natural and seminatural regions restricted the spread of Xf at the landscape scale. The results supported the hypothesis that the anthropogenic component of the epidemic played a key role. The obtained data also suggested the development of landscape-informed monitoring measures to stop the spread of Xf in Apulia and other Mediterranean nations. The investigation also emphasized the significance of explicitly taking into account the influences of the anthropogenic landscape when modeling Xf dispersion. Nevertheless, it becomes clear from the performed analyses that more research is needed on the main sources of uncertainty in the eco-epidemiological system under study. In particular, the susceptibility of the host plants, the rate at which the vector picks up the bacteria, the vector’s range of movement and the delay in disease detection should be considered.

Table 3 provides a summary of the key findings on the dynamics of Xf infection, a crucial component for the successful management of new foci.

**Table 3:** Studies involving the dynamics of geographical distribution of *Xylella fastidiosa* infection.

Authors and Year	Study Area	Method	Main Findings
Bosso et al., 2016 [91]	Italian territory between latitudes 45° N and 36° N and longitudes 6° E and 18° E	Maxent ver. 3.3.3k to model the potential distribution of Xf in Italy	Species distribution models showed a high probability of Xf occurrence in the regions of Apulia, Calabria, Basilicata, Sicily, Sardinia and coastal areas of Campania, Lazio and southern Tuscany
White et al., 2017 [93]	Apulia	Gompertz equation	The model highlights the importance of non-olive hosts which increase the spread rate of the disease and may lead to an order of magnitude increase in risk
Bucci, 2019 [92]	Apulian olive orchards	Analysis of the infection monitoring data set available by the Apulian regional government	Yearly epidemic spread 1–15 km from olive trees labeled as infected
White et al., 2020 [94]	Apulian olive groves	Epidemiological model	Through a Bayesian method, tree desiccation was estimated to occur approximately 4.3 years after symptom appearance
Cendoya et al., 2020 [96]	Apulia	Bayesian hierarchical models through the integrated nested Laplace approximation (INLA) methodology	This substantial contribution of the spatial effect in the models might indicate that the current extent of Xf in the study regions arose from a single focus or from several foci, which had coalesced
Castrignanò et al., 2021 [95]	An olive grove located in Oria (province of Brindisi, Southern Italy)	Data fusion procedure, based on non-parametric multivariate geostatistics	Remote and proximal sensor data with visual inspections and plant diagnostic tests provided a probabilistic map of Xf infection risk
Kottelenberg et al., 2021 [97]	Apulian olive groves	Deterministic model (a negative exponential function, a logistic function or a CNE function)	The model indicates that the disease spread started, approximately, in 2008 and the estimated rate of movement of the disease was 10.0 km per year
Gilioli et al., 2023 [98]	Apulian olive groves	Eco-epidemiological Model (XEM)	The model obtained described the infection dynamics of Xf outbreaks and showed that the abundance of the vector is the key factor determining the spread rate of the pathogen
Bajocco et al., 2023 [99]	Apulian olive groves	Ecological niche model	The anthropogenic component significantly contributed to the epidemic, with the road system representing the main driver of diffusion and natural/seminatural areas hampering Xf spread at the landscape scale

### 2.4. Diagnostic Methods

In the Salento area (Apulia Region, south-east Italy), where centenarian and millenarian plants represent a significant agronomic, economic and landscape trait as well as an important cultural heritage, Xf, and in particular the subspecies *pauca* (Xfp), have been reported as the causal agent of a devastating disease in olive trees. To lower the danger of infection for the nearby plants, it is crucial to create diagnostic methods that can identify the disease early, even when infected plants are still asymptomatic. Finding the source of an illness requires a diagnosis, and the investigation of the symptoms forms the basis of the diagnosis. The symptomatology might be non-specific if the alteration (symptom) can be attributed to multiple diseases or environmental factors or specific if the alteration (symptom) is typical of a particular pathogen. Serological and molecular approaches are the foundation of phytopathological diagnoses. In particular, the classical isolation on culture media and serological and molecular techniques are used in the diagnosis of Xf. An overview of the key findings in relation to the various diagnostic techniques used for Xf detection is reported in Table 4. The most sensitive assays were molecular, with no results left unclear, while direct tissue blot immunoassay (DTBIA) was more sensitive than double antibody sandwich–enzyme-linked immunosorbent assay (DAS-ELISA) among serological assays. The loop-mediated isothermal amplification (LAMP) technique [101] was taken into consideration as a viable alternative to PCR in the study by Yaseen et al. [102], due to its simplicity and high reliability. In this investigation, Xf in olive trees was discovered for the first time using this technique. Because the microorganism infects a variety of wild and domesticated plant species without manifesting any symptoms for varying periods of time, diagnosing Xf is challenging. According to estimates, the asymptomatic phase in the case of olive trees lasts for around 1.2 years [51,94,103]. The terminal leaf tip of the afflicted twigs exhibits the first sign of the ailment, which progresses inward and results in total desiccation, by turning from dark yellow to brown. These symptoms, however, signal an advanced stage of the infection that is irreversible and results in the death of the plant [12].

For each pathosystem, which is defined by the genotypes of the bacteria, host plant species, insect vector and environmental factors, Xf epidemiology and dynamics vary [104]. Different plant species can become infected by Xf and infections of susceptible hosts are known to cause xylem artery occlusions, to restrict water flow and consequently to cause the classic signs of desiccation. De Benedictis et al. [105] examined the xylem vessel occlusions in healthy and naturally infected *O. europaea* plants, grown in open fields, by investigating three common olive cultivars exhibiting different degrees of susceptibility to the disease. These included the susceptible cultivars “Ogliarola salentina” and “Cellina di Nardò” and the tolerant cultivar “Leccino”. Subject to infection, the most susceptible cultivars Ogliarola salentina and Cellina di Nardò showed a greater increase in occlusions than the more tolerant cv. Leccino. Moreover, occlusions were found to be caused by tyloses and gums/pectin gels and not directly by bacterial cell aggregates. Furthermore, as observed in Leccino plants, simple occlusion occurrence does not represent a direct marker of Xf tolerance/resistance, which could be rather related to a better capacity to regulate this response. “Cellina di Nardò” and “Ogliarola salentina” demonstrated high sensitivity to Xf also in the study of Luvisi et al. [27], whereas a notable resistance was noted in the less common cultivar “Leccino” [27,47,106]. In contrast to the severe dysbiosis seen in the Xf-susceptible cultivar “Cellina di Nardò”, their findings highlighted the stability of the endophytic bacterial microbiota in the leaves of the Xf-infected “Leccino” cultivar. This could imply that the preservation of a balanced microbiota and the presence of microorganisms specific to a particular cultivar could result in the “Leccino” resistance to Xf infection. Lignin deposition is a key component of the theories put forth to explain the putative tolerance of some hosts to Xf. Through the analysis of phenolic compounds in healthy and infected Leccino and Cellina di Nardò leaves, Sabella et al. [107] found that only in Leccino did the amount of quinic acid, a precursor to lignin, increase. On the other hand, a reduction of hydroxytyrosol glucoside, typically linked to drought and cold stress, was observed in both the investigated cultivars. In addition, histochemical examinations of stem sections revealed a different lignin distribution in the sclerenchyma and in the xylem tissue of infected Leccino plants compared to sections of healthy ones, suggesting a crucial role for lignin in the Xf tolerance of the Leccino cultivar.

Rey et al.’s research [108] aimed to achieve a flexible robotic-based strategy with proximal sensing tools designed specifically to detect Xf and suitable for deployment and to test it in the field. As a result, a small ground robotic platform called *X. fastidiosa*—Remotely Operated Vehicle for Infection Monitoring, or XF-ROVIM, was created for the early identification of Xf in various crops, primarily in olive trees. XF-ROVIM proved to be a versatile tool, affordable, simple to handle and capable of carrying remote or proximal sensing equipment, in order to inspect tree crops. It allows for the acquisition and storage of high-resolution field and geolocated data and can thoroughly investigate a field of 4 hectares without interruption in less than six hours. The obtained data made it possible to collect photos of every tree in the field from all four directions and to create field maps that display the 3D structure of the trees as well as several vegetative indices. Since the use of multivariate models that incorporate structural, geographical and spectral data is crucial in order to make an accurate forecast, the possible effective data gathering by XF-ROVIM at the field level could constitute a relevant achievement.

Moreover, olive tree infection by Xf may be related to element availability and content in the soil and host leaves. In the study by Scortichini et al. [109], soil and leaf samples were collected from 23 olive farms in Gallipoli (Lecce) and the surrounding areas that displayed symptoms of the disease. Utilizing inductively coupled plasma atomic emission spectroscopy, the amount of magnesium and other micronutrients was examined in each sample. Every tree was found to be Xfp-infected, according to results from real-time PCR. When compared to other places, soil and leaf samples from this area had lower micronutrient concentrations, as also demonstrated by multivariate data analysis. Widespread copper depletion found in infected leaves was unusual and had never before been observed in Italy, suggesting a possible diagnostic significance for the copper deficit in the olive leaves. In order to support olive production in the infected area, regional and national Italian institutions encouraged a series of actions for the replacement of old Xf-affected olive groves with new resistant varieties. These required further diagnostic evaluation and focuses. As already reported, a significant range of tolerance/resistance levels toward Xf was discovered in a number of local olive cultivars [20]. In this regard, for an optimal choice of possible replacements, Manici et al. [110] carried out a specific study with a focus on the interaction between soil physical characteristics and soil resident fungi, the microorganisms most involved in soil water retention and physical properties. Vergine et al. [111] also investigated and compared the autochthonous fungal and bacterial microbiota associated with both “Leccino” trees (Xf-resistant) and uninfected trees (Xf-uninfected) using the Xf-susceptible “Cellina di Nardò” trees as a control, in order to identify microbial taxa and parameters potentially involved in resistance mechanisms. The results also suggested that the maintenance of a healthy microbiota with higher diversity and the presence of cultivar-specific microbes might support the resistance of “Leccino” to Xf.

In order to quickly identify Xf symptoms in olive trees, large-scale monitoring techniques are necessary for successful strategies to eradicate and contain harmful pathogens. For the detection of infected hosts, even when they do not display visual symptoms, Zarco-Tejada et al. [112] used high-resolution hyperspectral and thermal imagery for two years to assess over 7000 olive trees, revealing an Xf infection months before symptoms became apparent to the human eye. As well, in 2020, Poblete et al. [113] used hyperspectral and thermal imagery collected during a two-year airborne campaign in a Xf-infected area to assess the performance of spectrally constrained machine-learning algorithms for this task. Their research demonstrated that large-scale Xf monitoring can be supported using airborne platforms carrying multispectral and thermal cameras. Di Nisio et al. [114] proposed image analysis of high-resolution visible and multispectral pictures captured by a multirotor unmanned aerial vehicle (UAV). The applied statistics showed that segmentation has a mean Srensen-Dice similarity coefficient of roughly 70% and that classifying affected trees has 98% sensitivity and 93% precision. Castrignanò et al. [115] also applied the use of a UAV, in conjunction with a multispectral radiometer, for the early identification of infection. Four drone flights, performed in three olive fields in the Apulia area of Italy between 2017 and 2019, allowed for the categorization of the Xfp severity level in olive trees at an early stage. Cross-validation showed that the non-parametric classification approach had an overall accuracy of 0.69 with a mean error rate of 0.31, while an accuracy of 0.77 and a misclassification probability of 0.23 were assessed for the early detection class.

With the aim of monitoring the incidence of Xf infection in olive orchards, Hornero et al. [116] investigated the suitability of Sentinel-2 satellite images for monitoring disease symptoms in Xf-infected olive orchards. Using field observations and multitemporal remote sensing data, their findings suggest that Sentinel-2 time-series imagery can provide useful spatiotemporal indicators to monitor the damage caused by Xf infections across large areas.

Understanding the chemical alterations caused by Xf colonization has been widely approached, using knowledge of the metabolic profile of infected olive trees. The metabolomics study, which is a bio-analytical tool used to explore complicated metabolic patterns linked with an organism’s response to physiological or pathological events (metabonomics), can be used to obtain this type of data. The first NMR-based metabolomic approach to study the metabolic effects of CoDiRO on local olive cultivars such as Ogliarola salentina and Cellina di Nardò was introduced by Girelli et al. [117]. More recently, an investigation conducted under carefully monitored circumstances contributes to the description of a group of compounds potentially acting as indicators in Xf infections in olive [118]. Two-year-old plants of the susceptible variety “Cellina di Nardò” inoculated with Xfp ST53 together with some xylem-inhabiting fungi in greenhouse conditions showed altered metabolics as demonstrated by NMR spectroscopy and MS spectrometry. Statistical analyses showed that *Xylella*-infected plants had higher levels of malic acid, formic acid, mannitol and sucrose than in *Xylella*-unaffected ones, but somewhat lower levels of oleuropein. Mannitol received attention as a possible marker, responsible for the olive tree’s ability to resist bacterial infection. In the study by Di Masi et al. [119], the difference between samples (leaf extracts) from healthy and infected olive trees was detected using an untargeted metabolomic method, using high-performance liquid chromatography coupled with quadrupole-time-of-flight high-resolution mass spectrometry (HPLC-ESI-Q-TOF-MS).

In 2021, Faino et al. [120] used the OXford Nanopore Technologies (ONT) MinION platform for detecting and identifying Xf at species, subspecies and sequence type (ST) levels. Their combined approach (“shotgun” strategy and Nanopore amplicon sequencing), which takes only a few hours, allowed for the detection and identification of Xf at the ST level in plant material with low bacterial infection.

Several metabolites were highlighted as potential specific biomarkers for the disease. In particular, a major dysregulation of several compounds from the flavonoid family led to the hypothesis that the host’s ability to defend itself decreases following Xf infection. Asteggiano et al.’s research [121] aimed to create a global metabolomics mass spectrometry assay for selecting OQDS molecular markers able to discriminate between healthy (HP) and infected (OP) olive tree leaves. The results of the multivariate analysis based on the HPLC-ESI HRMS platform clearly demonstrated the ability to distinguish the HP and OP samples. Interestingly, following the completion of numerous exploratory missions in the Apulia Region, Pavan et al. [122] also reported the discovery of 30 pauca symptomatic or asymptomatic plants in olive orchards that had been badly impacted by OQDS. This research, although also necessarily related to the performed disease diagnosis, essentially highlighted the constant task of finding cultivars resistant to OQDS. In particular, the genetic profiles of the investigated putatively resistant plants (PRPs), assessed by a selection of ten simple sequence repeat (SSR) markers, were compared with those of 141 Mediterranean cultivars. Interestingly most of the investigated PRPs formed a genetic cluster with 22 Italian cultivars, including “Leccino” and “FS17”, previously reported as resistant to Xf. The problem of quick and reliable monitoring of the infected area, allowing for an early diagnosis of the disease, even when the symptoms are not yet obvious, also constituted a constant key focus for the OQDS research. In order to discriminate between uninfected and infected but asymptomatic olive leaves with a sufficient degree of accuracy using hyperspectral data, Riefolo et al. [123] chose certain wavelengths suitable for a specific statistical analysis.

This method allowed for a useful preselection of samples suitable for further qPCR analysis, based on hyperspectral data investigation of olive leaves. D’Onghia et al. [124], with three subsequent studies, aimed to further improve sampling and testing for Xf-infected asymptomatic olive plants. Particularly, two out of the three considered studies focused on the temporal and spatial progression of Xf infections in the tree canopies of asymptomatic or slightly symptomatic olive trees of tolerant (Leccino) and sensitive (Cellina di Nardò and Ogliarola salentina) cultivars. All through the year, with the exception of the hottest and coldest months, sampling was most successful in the middle of the upper half of tree canopies. Using serological and molecular assays, the lower portions of mature leaves with petioles were less effective at detecting the pathogen than stem xylem tissues. Using stem xylem tissue as the most suitable matrix for testing, a third study was conducted based on these findings to compare molecular and serological tests (qPCR, real-time LAMP, DAS-ELISA, DTBIA) for the detection of Xf in the mid-upper part of asymptomatic branches of infected “Leccino” trees that were sampled in an appropriate collection time.

Using optical and transmission electron microscopy (TEM) simultaneously, Montilon et al. [125] investigated the frequency and distribution of vascular occlusions in the secondary xylem in stems of healthy or artificially infected Cellina di Nardò (susceptible) and Leccino (resistant) olives [126]. The study by Greco et al. [127] contains the first worldwide report of Xf subsp. *pauca* on Castanea sativa and the first characterization of Xf infection in this species. Asymptomatic Xf host species can play a considerable role in new outbreak emergence or in the expansion of existing ones.

The goal of Belmonte et al.’s work [128] was to examine the utility of unmanned aerial vehicle (UAV) photos in the battle against Xf. The considered information was a multiband UAV image taken on one occasion in an Xf-affected olive grove. Unmanned aerial vehicles have become increasingly popular in recent years, particularly for the management of plant pests. The research demonstrated the utility of UAV data in *Xylella* control, although there are still many issues to be resolved before autonomous detection of diseased plants in their early stages is possible. Easily accessible diagnostic methods also constitute a key focus in order to further improve the monitoring and containment of the disease progress. The work by Amoia et al. [129] devised a colorimetric LAMP technique employing as a template a crude alkaline sap (rather than purified total plant DNA) generated from the incubation of 50–60 mg of thin slices of olive twigs in a NaOH-containing buffer. This quick molecular assay, which takes 40 min from sample preparation to results, does not need a laboratory or any special tools and can be performed directly in the field, using just a portable isothermal block. Remote imaging data have been used not only for simple diagnosis but also for follow-up purposes, related to possible field treatments against Xf. Multi-resolution satellite data analysis has been recently used in the study by Blonda et al. [130] to assess the diagnostic efficiency of this technique and the results of specific treatments at both the field and tree scales. In order to evaluate plant conditions at the field level following treatments, High Resolution Sentinel-2 image data could be employed, while VHR imaging could be used to optimize treatment doses for each cultivar. According to the investigation, Ogliarola Salentina responded to treatments more favorably than Leccino and Cellina cultivars as also shown by in-field PCR and other results. Their findings suggested that Sentinel-2 time-series imagery can provide useful spatiotemporal indicators to monitor the damage caused by Xf infections across large areas. Savoia et al. [131] examined the variation in the reaction to Xf infection in a set of 100 autochthonous Apulian olive genotypes, encompassing reference cultivars, minor variants and F1 genotypes. The research made it possible to identify nine genotypes that are thought to be resistant to the disease. These genotypes comprise the initial panel of olive germplasm resources, which may be used as a reserve for replanting in affected areas as well as for researching the mechanisms of the pathogen.

Ciervo and Scortichini [132] systematically examined the eradicated plants that tested positive for Xfp, encompassing all asymptomatic ones within a radius of 100 or 50 m, over a 10-year period. The data show that the Xfp incidence in the “containment” and “buffer” zones is very low, especially during the last three campaigns from 2020–2021 to 2022–2023, when the bacterium was detected in a range of 0.06–0.70% of the sampled plants. Based on these data and according to epidemiological models that verified the negligible role of asymptomatic olive trees in the spreading of OQDS, it could eliminate the rule requiring the uprooting of all host plants that surround one Xfp-positive tree in a radius of 50 m in order to save many healthy centennial and monumental olive trees.

Table 4 provides an overview of the key findings in relation to the various diagnostic techniques.

**Table 4:** Studies based on the use of diagnostic systems with the application of various techniques.

Authors and Year	Methods	Aim of Diagnostic Tool	Main Findings
Yaseen et al., 2015 [102]	Real-time LAMP (real-time loop-mediated isothermal amplification)	Detecting on-site real-time Xf in host plants and insects	Real-time LAMP procedure displaying the advantages of an on-site detection method of easy handling, rapid execution and low cost
De Benedictis et al., 2017 [105]	Genotype identification and microscopy analysis	Identifying resistance mechanisms that can be exploited to prolong the productivity of olive orchards in the infected areas	They have shown that the interaction between Xf and *O. europaea*’s xylem vessels supports the indication of a secondary role for the pathogen in the occlusion process, where symptoms were correlated to a physiological response of the plant
Luvisi et al., 2017 [27]	Molecular analysis	Selecting markers to determine whether or not there is symptom progression	Differences in the induced responses of quinic acid among four olive cultivars (Cellina di Nardò, Ogliarola di Lecce, Frantoio and Leccino) suggest that they play defensive roles in olive tree response to Xf infection
Girelli et al., 2017 [117]	NMR spectroscopy	Metabonomics to explore complicated metabolic patterns linked with an organism’s response to physiological or pathological events	The changes in the metabolomic profiles obtained from Ogliarola salentina and Cellina di Nardò are reported upon the DENTAMET^®^ treatments
Sabella et al., 2018 [107]	Molecular and phenolic analyses	Understanding the processes contributing to the putative Xf tolerance of olive trees cv. Leccino	Results suggest a critical role for lignin in Xf tolerance of cv. Leccino, since the quantification of lignin in healthy and infected branches of both cultivars showed a significant increase in total lignin in infected Leccino compared with that in the sensitive cultivar
Rey et al., 2018 [108]	Sensing technologies and field tests	Mapping the distribution of plant diseases for early detection of Xf in olive groves at plant to leaf levels	They developed a small low-cost field robot capable of inspecting the whole field continuously, capturing geolocated spectral information and the structure of the trees for later comparison with the in situ observations
Scortichini et al., 2019 [109]	Real-time PCR and inductively coupled plasma atomic emission spectroscopy (ICP-AES)	Management strategy aiming at reducing the spread of the bacterium through the study of the element content and availability in the soil and in the host leaves	They indicated that Xf. subsp. *pauca* infection causes a depletion of copper within olive leaves
Sabella et al., 2019 [103]	SEM-EDX analysis and molecular analysis	Evaluating cavitation susceptibility and activation of refilling mechanisms to restore hydraulic conductivity in olive plants subjected to Xf infection	They indicated that resulted gene expression patterns suggested that the infected plants of the cultivar Leccino strongly modulate the genes involved in embolism sensing and refilling
Manici et al., 2019 [110]	Molecular analysis (DGGE analysis)	Improving the decisional tools for selecting disease-decimated groves to be replaced with new olive trees	Multiple correlation and canonical correspondence analyses led to identification of a series of soil physical and fungal indicators, which were linearly correlated with the Xf-infected area
Vergine et al., 2019 [111]	Molecular and sequence analysis	Improving biological treatment of OQDS	The maintenance of a healthy microbiota with higher diversity and the presence of cultivar-specific microbes might support the resistance of “Leccino” to Xf
Castrignanò et al., 2020 [115]	Unmanned aerial vehicle (UAV) with a multispectral radiometer	Early detection of infection	The results encourage the application of UAV technology for the early detection of Xf infection
Di Nisio et al., 2020 [114]	Unmanned aerial vehicle (UAV) with multispectral imaging	Early detection of infection	Image processing of high-resolution visible and multispectral images acquired by a purposely equipped multirotor unmanned aerial vehicle (UAV) is proposed for fast detection of Xf symptoms in olive trees
Hornero et al., 2020 [116]	3D radiative transfer modelling (3D-RTM) and Sentinel-2 satellite data	Early detection of infection	This study emphasizes the value of detecting anomalies in vegetation health by interpreting temporal variations in model retrievals
Poblete et al., 2020 [113]	Hyperspectral and thermal imagery	Early detection of infected hosts	Results of this study demonstrate that multispectral and thermal cameras can be used for large-scale monitoring of Xf-infected areas
Asteggiano et al., 2021 [121]	Liquid chromatography separation and high-resolution mass spectrometry detection	Identification of molecular markers to discriminate between healthy and infected olive tree leaves	Results obtained via multivariate analysis through an HPLC-ESI HRMS platform show a clear separation between healthy and infected samples
Faino et al., 2021 [120]	OXford Nanopore Technologies (ONT) MinION platform	Detection and identification of Xf from infected plant material	The results pave the way for novel opportunities for Nanopore sequencing as an effective surveillance tool for early detection of Xf
Jlilat et al., 2021 [118]	NMR spectroscopy and MS spectrometry with a non-targeted approach	Describing a set of metabolites playing a possible role as markers in the infections by Xf in olives	This study revealed that *Xylella*-infected plants were characterized by higher amounts of malic acid, formic acid, mannitol and sucrose than in Xf-non-infected ones
Riefolo et al., 2021 [123]	Real-time PCR, hyperspectral analysis and partial least square regression (PLSR)	Improving diagnostic assessment of plants and evaluation of their phytosanitary status	Using only spectral data, it is possible to discriminate the infected plants at a very early stage of infection, saving time and financial resources
Pavan et al., 2021 [122]	Molecular analysis	Identifying genotypes putatively resistant to Xf	Some of the putatively resistant plants (Leccino) identified in this study might be exploited in cultivation or as parental clones of breeding programs
D’Onghia et al., 2022 [124]	Molecular and serological tests (qPCR, real-time LAMP, DAS-ELISA, DTBIA)	Early detection of infection	This study was conducted to compare molecular and serological tests for detection of Xf using stem xylem tissue as the most appropriate matrix for testing
Di Masi et al., 2022 [119]	High-performance liquid chromatography and quadrupole-time-of-flight high-resolution mass spectrometry (HPLCESI-Q-TOF-MS)	Discovering disease-associated biomarkers in plants	This study suggests there is a decrease in the defense capabilities of the host after Xf infection due to a significant dysregulation of some metabolites belonging to the flavonoid family
Montilon et al., 2022 [125]	Electron microscopy analysis	Early detection of infection	Their study suggests that exploitation of pit membranes is a key event in the infection process of Xf subsp. *pauca* ST53 in susceptible olive cultivars
Amoia et al., 2023 [129]	Isothermal amplification and colorimetric LAMP	Improving the monitoring and containment of the disease spread	A portable, sensitive and target-specific Xf field test was developed, which has a 40 min sample-to-answer time and does not require any DNA isolation procedure or laboratory equipment
Belmonte et al., 2023 [128]	Unmanned aerial vehicles (UAVs)	Implementing the automatic detection of infected plants in the early stages	The work has shown the potential of data from unmanned aerial vehicles in *Xylella* control, but many problems still need to be solved for the automatic detection of infected plants in the early stages
Blonda et al., 2023 [130]	High-resolution (HR) Sentinel-2 images and very-high-resolution (VHR) Pléiades images	Implementing the automatic detection of infected plants in the early stages	HR image data could be used to evaluate plant conditions at field level after treatments, while VHR imagery could be used to optimize treatment doses per cultivar
Greco et al., 2023 [127]	Molecular test (MLST) and phylogenetic analysis, FISH (fluorescence in situ hybridization) together with a CLSM analysis (confocal laser scanning microscope)	A diagnostic investigation for Xf is carried out on several small groups of chestnut trees in Salento, coupled with the observation of any visible symptoms	This work shows how knowledge of all host species, including the pauci-symptomatic and asymptomatic ones, and of the vectors present in a given area, is essential to make containment measures truly effective
Savoia et al., 2023 [131]	Molecular test (qPCR, genotyping)	Search for new genotypes that are tolerant or resistant to Xf	Identification of nine putatively resistant genotypes that represent the first panel of olive germplasm resources
Ciervo and Scortichini, 2023 [132]	Molecular and serological tests (PCR, ELISA)	Eliminate the rule requiring the uprooting of all host plants that surround one Xfp-positive tree in a radius of 50 m	They suggested that the rule requiring the uprooting of all host plants that surround one Xfp-positive tree in a radius of 50 m in the “containment” and “buffer” zones could be eliminated

### 2.5. Treatments

Since Apulia is constantly the first olive oil producer region in Italy, the Apulian and Salento olive oil industries, in particular, have been severely impacted by the Xf bacterium’s widespread distribution. Since its discovery in 2013, phytosanitary controls and financial interventions have been gradually put in place to battle this pathogen and support business owners in the industry and the affected territories. Priorities addressed in the action plan in response to the discovery of the quarantine bacterium included the creation and deployment of a monitoring program that could precisely define the area affected by Xf infections. The epidemiological expertise gained in the United States and Brazil, characterized by other Xf bacterial strains well-established for some time, as well as the experience gained in the Apulian territory against other quarantine pathogens were essential in achieving this goal. In addition to their economic importance, olive trees in Apulia (many of them being even over 1000 years old) are also preserved as local heritage and regarded as symbols of the region’s character due to their widespread distribution. Interestingly, an olive tree is also present on the coat of arms of the Apulia region.

The above should have suggested a specific consideration for all possible conservative approaches when putting a stop to the spread of the disease, despite the statutory prescriptions expected for a quarantine pathogen. On the other hand, the Xf tentative treatment attempts both in controlled (green house and in vitro) and open field conditions (whenever possible) remain very limited and are essentially represented by the literature reported below and summarized in Table 5. Girelli et al. [117] examined the effects of CoDiRO on symptomatic olive trees using an NMR-based metabolomics approach. In this study, the effects of Dentamet^®^ [133], a CE-approved fertilizer sprayed on Salento olive trees with symptoms, were examined. Dentamet^®^ contains a combination of zinc and copper complexes with hydracids of citric acid. This patented foliar fertilizer has a specific weight of 1.280 g/L and a zinc and copper content of 4% and 2%, and it can be viewed as a product with a dual action formulation. According to its technical sheet, this biocomplex, which quickly corrects Zn and Cu deficiencies, also induces resistance for the chemical structure is similar to those of several anti-microbial substances released from the plant in response to various stresses. Dentamet^®^ is approved for use in organic farming and is reportedly sold in more than 30 countries across the world due to its few negative effects on the environment and few usage restrictions. The metabolomic profiles of aqueous extracts, from the leaves of the two olive cultivars Ogliarola Salentina and Cellina di Nardò, changed after Dentamet^®^ treatments. In particular, different and opposite polyphenolic and sugar patterns in the two cultivars, which showed a different incidence and severity of disease before the treatments, were observed after treatments.

Metabolomics, usually based on mass spectrometry (MS) and/or nuclear magnetic resonance (NMR) and multivariate data analyses, provides an overview of all detectable small metabolites characterizing a specific substrate. Metabolomics is one of the analytical methodologies available for deciphering molecules involved in the plant–microbe interaction, allowing specific useful comparisons (infected vs. non infected; treated vs. non treated, etc.). Therefore, as a key combination of NMR and chemometrics, the metabolomics approach may be helpful in order to define biological processes in plants under stress conditions, such as pathogen infections [134]. This understanding of plant physiology is essential for the development of applications in disease prevention and treatment monitoring. Plant micronutrients and secondary metabolites, such as phenolic compounds, would appear to play significant roles in the disease infection process induced by Xf in numerous crops.

Scortichini et al. [135] further tested the zinc–copper–citric acid based biocomplex Dentamet^®^ in a three-year field experiment on Ogliarola salentina and Cellina di Nardò orchards, naturally infected by the bacterium. In vitro and in planta bactericidal activities were also investigated.

From June 2016 to September 2017, quantitative real-time PCR experiments were conducted in accordance with the standard protocols set forth by the European and Mediterranean Plant Protection Organization. The results showed a statistically significant decrease in the Xfp cell densities in the leaves of treated trees. Six spray treatments of Dentamet^®^ 0.5% (*v*:*v*) were administered to the olive tree crowns every year from early April to October (apart from July and August). In both cultivars, the compound lessened the severity of symptoms. By the end of the study, the majority of untreated trees had perished, but all treated trees had thrived, as determined by a normalized differential vegetation index. These encouraging findings suggested that integrated management to reduce Xfp severity, including frequent soil harrowing and pruning, together with spring and summer Dentamet^®^ spray treatments, could likely successfully control the disease. Girelli et al. in 2019 [136], using a ^1^H NMR-based metabolomic method, performed a comparison of the Xfp-infected Ogliarola Salentina and Cellina di Nardò with trees that had undergone the zinc–copper–citric acid biocomplex, over a year of treatment. By comparing the metabolomic patterns of leaf samples taken from untreated and treated trees of the two olive cultivars, potential biomarkers of the disease were also found. Some metabolites, including quinic acid, oleuropein in its aldehydic form, ligstroside and phenolic compounds, were consistently identified in both cultivars as being discriminative for the Dentamet^®^-treated versus untreated infected olive trees. Bleve et al. [137] tested in vitro, for the first time, the selected bacterium Xf Salento-1 (NCCPB No. 4595 LMG 29352) [138], representative of the outbreak strain causing OQDS in Apulia. The study aimed at determining its susceptibility/resistance to various antibiotics, phenols, fungal toxins and crude culture extracts. Among the investigated substances, 17 out of the 32 tested antibiotics did not have an impact on bacterial growth at a dose of 5 μg/disk, according to a bioassay based on the agar disk diffusion method. The findings of this study are the first to describe some of the chemotypic characteristics of the outbreak strain of Xfp (phylotype ST53) that causes OQDS in Apulia. The nutrients found in the soil and leaves may be connected to other facets of OQDS epidemiology. Assessing the physiological status of the plant in connection to the pathogen infection can also benefit from the examination of the full profile of the mineral nutrients and trace elements. Del Coco et al. [139] evaluated the micronutrient content along with calcium, sodium and magnesium in the soil and leaves of olive groves in various areas of Apulia and the neighboring Basilicata region in order to evaluate differences in soil and leaf ionome composition between olive groves of the “infected” and “free” areas of Apulia. The soil and leaf ionomic composition of the olive farms growing north of the Salento Barletta-Andria-Trani (BAT, Apulia) and Potenza (PZ, Basilicata, Apulia bordering region) provinces were significantly different from that of the infected olive groves of the Salento areas (LE, BR, TA provinces). Moreover, the Dentamet^®^ biocomplex-treated trees in the afflicted Salento districts had a noticeably different leaf ionomic profile than the untreated plants. Compared to untreated, treated trees had leaves with a higher zinc concentration. Interestingly, Leccino, a *Xylella*-resistant tree, was characterized by a greater manganese concentration than the more pathogen-sensitive Ogliarola salentina and Cellina di Nardò among the uninfected trees. Zicca et al.’s study [140] sought to identify antagonistic microorganisms that might be used as biocontrol agents against Xf by using essentially antagonistic activity testing methods.

Endophytic bacteria, obtained from olive trees affected with the disease but exhibiting only minor symptoms, as well as Bacillus strains, previously recognized as biocontrol agents, were the two antagonistic microorganisms investigated. It was also discovered that the majority of the endophytic bacterial isolates belonged to various species of the genera *Sphingomonas*, *Methylobacterium*, *Micrococcus* and *Curtobacterium*. However, none of them showed antagonistic activity when they were tested in vitro against Xf-ST53. Contrarily, striking antagonistic activities were seen when isolates from several species of the genus Bacillus were included in these assays. A few *B. velezensis* strains also generated culture filtrates with anti-Xf-ST53 inhibitory activity. Liccardo et al. [141] put forth, in their study, a biological alternative to chemical control action suggesting the possible use of *Zelus renardii*, a natural enemy of Ps, for adult vector population and infection biocontrol. They demonstrated that a *Z. renardii* inundation strategy has the potential to provide an effective and “green” solution to Xf invasion, with a reduction in the pathogen incidence below 10%.

In order to develop a sustainable phytopathogen control research, which can offer a targeted solution against Xf, reducing side effects on environment and human health, Baldassarre et al. [142] proposed a nanotechnological approach. They obtained a new formulation of the systemic fungicide, Fosetyl-Al Nanocrystals, employing ultrasonication assisted production of water-dispersible nanocrystals. This formulation with chitosan as a coating agent resulted to be very stable over time, less toxic compared to conventional formulation and with an interesting antibacterial activity against two Xf subspecies.

Tatulli et al. [143] further assessed the in vitro and in planta mid-term effectiveness of Dentamet^®^, towards one strain of Xfp, in order to provide a control strategy aimed at maintaining the tree productivity in the infected areas. The substance had in vitro bactericidal action and prevented the growth of biofilms in a number of typical strains of the Xf subspecies, including the Apulian strain Xfp (ST53), which was isolated from olive trees in Apulia. The regular spraying of the biocomplex over three or four consecutive years resulted in a low concentration of Xfp over the seasons, according to the field mid-term evaluation of the control approach measured by quantitative real-time PCR in 41 trees of two olive groves in the “infected” area. After six consecutive treatments, the bacterial concentration, in particular, decreased from March to July to October. In a ^1^H NMR metabolomics study, Girelli et al. [144] also focused on a mid-term comparison of the Dentamet^®^ treatment effects including the Leccino cultivar, which is known to show tolerance to the disease progression. The overall metabolism of the tolerant cultivar Leccino in comparison to the susceptible cultivars Ogliarola salentina and Cellina di Nardò under periodic mid-term Dentamet^®^ administration was investigated during a multiyear on-field usage schedule (6 months treatment April–September after 6 months suspension). The metabolic profiles of each cultivar were examined with a focus on the two samplings (March, prior to the start of treatments, and October, following their conclusion). The findings showed that the tolerant Leccino cultivar had a more targeted metabolic response than sensitive cultivars. In particular, Leccino showed a significant difference between treated and untreated samples in the first sampling, indicating a potential memory impact of treatment from prior years that was unique to this cultivar. In their study, Bruno et al. [145] first documented the effects of NuovOlivo^®^ in two severely OQDS-affected olive fields in the province of Lecce. Natural detergent NuovOlivo^®^ (Ministero dello Sviluppo Economico patent no. 102017000109094, Italy) is manufactured from plant oils and extracts of numerous botanical species, along with sodium and calcium hydroxide, sulfur and sodium bicarbonate, which works as an activator. Olive plants, treated and untreated, were compared regarding Xfp density using quantitative real-time PCR. In the initial study, olive trees from Montesano Salentino’s Cellina di Nardò, between 70 and 75 years old, were used. Here, after seven treatments, the plants started to develop drupe fruits, and the McKinney’s disease index percentage dropped to 2.5%. Twice a year, between March and October, olive trees were treated, and they were also pruned in the winter. The second experiment focused on 60- to 65-year-old olive trees, growing in Sternatia (Lecce province), with a disease score of 90.88%, which fell to 4.0% after three spray treatments over two years. According to qRT-PCR, the treated trees in both olive cultivars had decreased Xfp DNA concentrations, also suggesting NuovOlivo^®^ as a possible curative product restricting and/or stopping the damaging epidemic. Specific olive grove sustainable management has also been suggested in order to improve the ecosystem balance and plant resistance to environmental shocks. Although not specifically focused on Xf-infected plants, Fausto et al. [146] conducted trials in two olive groves in the Salento infected area, one under organic agricultural practice and the other managed conventionally (controls), both of which were subsequently converted to sustainable management (i.e., regular light pruning, soil and foliar fertilization and cover crops). A metabolomic analysis of the major metabolites of the xylem sap using untargeted gas chromatography mass spectrometry (GC-MS) suggested that the adoption of sustainable agronomic practices could increase both the resistance and resilience to biotic and abiotic stresses in this important tree crop. Subsequently, Scala et al. [147] used supervised learning algorithms to classify infected Salento olive tree samples from the cultivars Ogliarola salentina and Leccino, treated or untreated with Dentamet©, using high-performance liquid chromatography-mass spectrometry-MS-based targeted lipidomics. Focusing on xylem tissue lipidic extracts analysis, the efficacy of the Cu-Zn biocomplex treatment in controlling the disease was further assessed. It was also hypothesized that specific lipid substances such as oxylipins may be employed as biochemical diagnostic markers to distinguish between damaged and healthy plants. Ambrico et al. [148] looked into the effectiveness of low-temperature plasma and plasma-activated water to kill bacterial cells as a potential remedy. In vitro tests were performed to determine whether directly applying a surface dielectric barrier discharge (SDBD) plasma to bacterial cells and plasma-activated water (PAW) would have a biocidal impact. According to the findings, a high decontamination rate was obtained even for Xfp cells buried in biofilms cultured on solid media, and a liquid culture medium completely rendered the cells inactive. The effects of Dentamet© on endotherapy-treated plants were examined in the study by Girelli et al. [149], which was the first report on a metabolomic investigation of endotherapy-treated Xfp-infected olive trees. In this study, the researchers used a combined approach to examine the effects of Dentamet© for infected Ogliarola salentina and Cima di Melfi olive trees after precision intravascular biocomplex delivery using a novel injection system. The investigators noticed particular differences in the leaf content of certain metabolites that could be followed in their evolution after endotherapy treatment allowing a detailed time course analysis of in planta Dentamet© effects. In particular, the method revealed that quinic acid, a disease biomarker, and mannitol significantly decreased after the injection, while associated molecules to polyphenols and oleuropein increased. The overall endotherapy effects, at the used doses, reached a maximum after 2 days from the injection to slowly completely smooth down after two weeks, offering useful indications for possible treatment schedule optimization.

New nanocarrier-based agrochemical tools have also been investigated to effectively protect plants from quarantine infections. These include biocides such as aromatic secondary metabolites naturally found in plant extracts, essential oils and gels. Baldassarre et al. [150], using both a fluorometric assay and an in vitro inhibition assay, published the first investigation of the impact of thymol on Xfp. This study demonstrated the powerful antibacterial properties due to synergistic interaction between thymol and CaCO_3_ nanocarriers, suggesting the possible use of these agrochemical formulations as efficient biocides to control Xfp infection.

Hussain et al. [151] reported the medium-term effects of foliar spray and endotherapy treatments with varying doses of Dentamet^®^ in Xfp-infected 150-year-old olive trees of Ogliarola salentina and Cellina di Nardò cultivars, studied by ^1^H NMR-based metabolomics. Investigators compared the outcomes at 60, 120 and 180 days to those of a conventional foliar spray therapy and a water injection used as a control. The analysis of the metabolic profile revealed alterations in plant metabolites linked to the development of the disease, such as chemicals associated with mannitol, quinic acid and oleuropein. Monthly endotherapy sessions produced the best outcomes in terms of separating the metabolic profiles with regard to water injection. In comparison to intravascular treatments, Dentamet^®^ foliar application showed more precise time-related progressive effectiveness. Therefore, in addition to the potential for endotherapy to be more successful than foliar treatments, the requirement for additional doses/frequencies trimming to achieve long-term outcomes was also evaluated. In conclusion, the current field investigations supported the findings that some agrochemicals such as Dentamet^®^ may be efficient in inducing metabolic variation, which may be related to a reduction in the onset of symptoms for olive quick decline syndrome (OQDS) associated with Xfp. On the other hand, at present, there are strict eradication and containment requirements because there is no specific therapeutic indication for this infection and because Xfp is classified as a regulated quarantine pathogen in many nations across the world. According to the current EU law, containment procedures are in force in the regions where the bacterium is well-established, like the Apulia region, requiring drastic containment techniques to reduce and manage infections. In order to determine whether pruning interventions could reduce the systemic spread of the bacterium and the severity of the desiccation phenomena, Camposeo et al. [152] set up a field trial. The removal of all the symptomatic branches and significant or minor pruning operations on the highly susceptible cultivars, such as Cellina di Nardò trees, did not result in a decreased bacterial colonization or symptom development. No discernible improvement in the health status of the infected olive trees was observed after two years of focused pruning treatments, indicating that their sole application is not enough to mitigate the bacterium’s effects on the sensitive olive trees. On the other hand, an endotherapeutic experiment employing a phenolic extract from olive leaves, to combat the bacterium in naturally infected mature olive trees, produced excellent results, according to Vizzarri et al. [153]. The use of phenolic extracts also gave better results in comparison to analogous treatments based on garlic powder and potassium phosphite. Polar phenols were proven to be statistically beneficial in the two years of in-plant testing in promoting the vegetative growth of the treated trees, most likely as a result of the bacteriostatic action assessed in vitro for both treatments. Nevertheless, according to the authors, the experiments conducted in their study proved to be unsuitable for centenary trees. The study by Orfei et al. [154] aimed to evaluate the feasibility of protecting plants against attacks of gram-negative and gram-positive phytopathogenic bacteria by using electrochemically synthesized silver ultra-nanoclusters named ARGIRIUM-SUNCs^®^. This compound inhibited the in vitro growth and biofilm formation of *Xylella fastidiosa* subsp. *pauca*, *Pseudomonas syringae* pv. tomato, *Xanthomonas vesicatoria* and *Clavibacter michiganensis* subsp. *michiganensis*. Therefore, treatments with ARGIRIUM-SUNCs are a possible alternative control measure.

Table 5 summarizes the key findings in relation to the treatments attempts for Xf infection management.

**Table 5:** In vitro assays and studies based on treatments of infected plants.

Authors and Year	Methods	Treatment Type	Main Findings
Girelli et al., 2017 [117]	NMR spectroscopy	On field with active principle—Dentamet^®^, a CE-approved fertilizer containing zinc, copper and citric acid	In this study, the changes in the metabolomic profiles obtained from leaves of Ogliarola salentina and Cellina di Nardò are reported
Scortichini et al., 2018 [135]	Molecular analysis (quantitative real-time PCR), confocal laser scanning microscopy, fluorescent quantification and atomic emission spectroscopy	On field with active principle—Dentamet^®^, a CE-approved fertilizer containing zinc, copper and citric acid	In this study, a 3-year field trial with Dentamet^®^ in an olive orchard containing Cellina di Nardò and Ogliarola salentina olive trees was carried out. The data revealed a statistically significant reduction of Xf cell densities within the leaves of treated trees
Bleve et al., 2018 [137]	HPLC-DAD analysis and agar disk diffusion method	In vitro with agar disk diffusion method	In this study, in vitro antimicrobial activities of different classes of compounds against ST53 were evaluated
Girelli et al., 2019 [136]	NMR spectroscopy	On field with active principle—Dentamet^®^, a CE-approved fertilizer containing zinc, copper and citric acid	A consistent increase in malic acid was observed for the Ogliarola salentina trees, whereas in the Cellina di Nardò trees, the treatments attenuate the metabolic response to the infection
Baldassarre et al., 2020 [142]	Toxicological study and antibacterial activity	In vitro characterization and analysis of Fosetyl-Al Nanocrystals	Results of in vitro assays (dosage and administration modality) suggest the possible use of this new nanoformulation in an integrated pest management strategy for an in-filed control of Xfp
Del Coco et al., 2020 [139]	Inductively coupled plasma atomic emission spectroscopy (ICP-AES) and molecular analysis (real-time PCR)	On field with active principle—Dentamet^®^, a CE-approved fertilizer containing zinc, copper and citric acid	They observed that soil and leaf ionomic composition of olive farms growing in the pathogen-free areas north of the Salento and Potenza provinces is significantly different from that shown by the infected olive groves of the Salento areas
Liccardo et al., 2020 [141]	Numerical simulation	In vitro	They proposed an available natural enemy of *Philaenus spumarius*, i.e., *Zelus renardii,* for adult vector population and infection biocontrol, with a reduction in the pathogen incidence below 10%
Zicca et al., 2020 [140]	Molecular analysis	In vitro	The identification of antagonistic bacteria potentially deployable as biocontrol agents against Xf
Tatulli et al., 2021 [143]	In vitro antibacterial assays and molecular analysis (real-time PCR and qRT-PCR)	On field with active principle—Dentamet^®^, a CE-approved fertilizer containing zinc, copper and citric acid	The compound showed in vitro bactericidal activity and inhibited the biofilm formation in representative strains of Xf on field, in the Xf-sensitive cultivars Ogliarola salentina and Cellina di Nardò or in the Xfp-resistant Leccino
Bruno et al., 2021 [145]	Molecular analysis (PCR and quantitative real-time PCR)	On field with active principle—NuovOlivo^®^, a natural detergent made from plants oils and extracts of multi botanical species plus sodium and calcium hydroxide and sulphur, activated with sodium bicarbonate	The leaves of treated plants showed a low total phenolic content and no cell membrane damage associated with lipid peroxidation and electrolyte leakage; therefore, NuovOlivo^®^ works as a curative product limiting and/or stopping the destructive epidemic caused by this bacterium
Fausto et al., 2021 [146]	Gas chromatography–mass spectrometry	Trials were carried out, in vitro, in two olive groves, one organically and one conventionally managed (controls), successively both converted to sustainable management	Many of the compounds with increased levels under sustainable management have a well-known role as osmoprotectants or are involved in plant defense
Girelli et al., 2021 [144]	NMR spectroscopy	On field with active principle—Dentamet^®^, a CE-approved fertilizer containing zinc, copper and citric acid	They highlighted a specificity in the metabolic response of the tolerant Leccino compared to susceptible cultivars
Scala et al., 2022 [147]	High-performance liquid chromatography–mass spectrometry–MS-based targeted lipidomics with supervised learning algorithms	On field with active principle—DENTAMET^®^, a CE-approved fertilizer containing zinc, copper and citric acid	They built classifiers using the relative differences in lipid species able to discriminate olive tree samples, infected and non-infected, belonging to different cultivars, and treated or untreated with DENTAMET^®^
Camposeo et al., 2022 [152]	Molecular analysis (quantitative real-time PCR)	On field with physical treatment—pruning	They set up a field trial to assess if pruning interventions could limit and/or recover Xf-infected trees by reducing the systemic spread of the bacterium but, no significant amelioration of the sanitary status of the infected olive trees was recorded
Ambrico et al., 2022 [148]	Emission spectroscopy and direct application of a surface dielectric barrier discharge (SDBD) plasma on bacteria cells and plasma-activated water (PAW)	In vitro to test the biocidal effect of the direct application of SDBD plasma on bacteria cells and PAW	They investigated the efficacy of the low-temperature plasma and plasma-activated water to kill bacterial cells and the results showed a high decontamination rate even for cells of Xf embedded in biofilms
Girelli et al., 2022 [149]	Agro-active endo-therapy and NMR spectroscopy	On field with active principle—Dentamet^®^, a CE-approved fertilizer containing zinc, copper and citric acid	Metabolomics approach showed a significant decrease in both the disease biomarker with simultaneous increase in polyphenols and oleuropein in Ogliarola salentina and Cima di Melfi olive trees
Baldassarre et al., 2023 [150]	In vitro inhibition assay	In vitro	They reported the great antibacterial effect of thymol on Xf, suggesting the potential application of thymol-nanoparticles as effective biocides to control Xf infection
Hussain et al., 2023 [151]	Trunk injection and foliar spray treatments of infected olive trees and NMR spectroscopy	On field with active principle—Dentamet^®^, a CE-approved fertilizer containing zinc, copper and citric acid	They reported the medium-term effects of foliar spray and endotherapy treatments with different doses of Dentamet^®^ in Xf-infected olive trees cvs Ogliarola salentina and Cellina di Nardò and the best results were found for monthly endotherapy treatments
Vizzarri et al., 2023 [153]	Endotherapeutic trial using a phenolic extract in comparison with a solution based on garlic powder and potassium phosphite	In vitro with a solution based on garlic powder and potassium phosphite	The use of phenolic extracts also gave better results in comparison to analogous treatments based on garlic powder and potassium phosphite
Orfei et al., 2023 [154]	In vitro antimicrobial activity and effect on bacterial biofilm formation and disruption	In vitro with electrochemically synthesized silver ultra nanoclusters ARGIRIUM-SUNCs^®^	ARGIRIUM-SUNC has strong antimicrobial activities against phytopathogenic bacteria and inhibits biofilm formation at low doses

### 2.6. Impacts on the Environment, Man and Society

One of the most fruitful areas of Apulian agriculture is the olive industry. With an average yearly production of roughly 315,000 tons of olive oil, Apulia stands out as the top region in the country [155]. According to statistical information on the production of olive oil, the years immediately following the appearance of Xf were particularly detrimental. According to data analysis from the SIAN (National Agricultural Information System), oil production in Lecce specifically had a negative trend in the 2019–2020 campaign, with a decline of 80% and an all-time low of 3979 tons produced. ISMEA [156] production data show a 20% recovery for the 2023/24 campaign, despite a tough year characterized by a winter drought followed by spring rains that hampered fruit setting in several places. According to the statistics collected, production is over 290 thousand tons, up 20% from last year. For the first time, the Apulia region will produce more than 60% of the nation’s olive oil. In their study, Schneider et al. [157] simulated the future spread of the disease based on climatic-suitability modeling and radial expansion of the invaded territory. For Italy, across the considered rates of radial range expansion the potential economic impact over 50 y ranges from 1.9–5.2 billion Euros for the economic worst-case scenario, in which production ceases after the orchards die off.

The phytosanitary emergency has effects on the environment, society and the well-being of the local inhabitants in addition to the typical economic and productive aspects [25,29].

A high standard of living for humans depends on the health of ecosystems and the processes that take place in them. Environmental factors, in particular, play a significant role in determining health, and human well-being is correlated with the ecosystem function and their condition of excellent conservation. The so-called “ecosystem services” that ecosystems offer to man are all those products and services that either directly or indirectly meet his requirements. The Xf outbreak has had an impact on a number of ecosystem functions that olive groves provide [158].

Due to the death, degradation and slowed development of olive trees as well as the difficulty of replacing susceptible cultivars quickly, there has been a major reduction in the production of table olives and olive oil, both numerically and qualitatively. As previously indicated, one of the most promising methods to lessen the long-term damage in regions where the bacterium is currently endemic is the replanting of partially resistant olive cultivars (such as Leccino and Favolosa). However, because it takes several years for the newly planted trees to become productive (5 to 20, respectively, for intensive and conventional farming methods), their replanting does not address the short-term supply issue.

On the other hand, Ogliarola salentina and Cellina di Nardo, two local cultivars, are also major constituents of a PDO product (Terra d’Otranto EVOO) well-characterized [159,160] and very well-known, thanks to the touristic importance of the area. Due to their susceptibility to *Xylella*, there is a very significant risk of losing these cultivars and subsequently the health and economic advantages associated with them. Mathematical models are crucial tools that enable a quantitative examination of an epidemic system and the subsequent identification of potential control or even prevention techniques. The model developed by Brunetti et al. [161] revealed threshold parameters as possible targets of control measures within the framework for integrated pest management. This is without losing the productive resource represented by the olive trees. The results of the mathematical analysis, supported by numerical simulations, suggested the removal of an appropriate amount of weed biomass, constituting a reservoir for Xf in olive orchards and the surrounding areas, as the most effective method for preventing the spread of OQDS. Additionally, even though less economical, the analysis demonstrated the use of more resistant cultivars as a successful technique for pathogen control. Specific work demonstrated that Xf infestation also affects the local climate. In the summer, when temperatures are at their greatest, olive orchards are particularly useful in terms of climate mitigation. As a result, the absence of olive trees raises soil temperature, causes more evaporation and reduces CO_2_ sequestration. In the long run, however, these detrimental effects might be reduced by replanting alternative tree species. The disease, in its most severe forms, could result in a temperature increase of about 2 °C compared to the pre-*Xylella* evaluation. According to Semeraro et al.’s [162] evaluation of ground temperature following the olive tree loss, this effect reduces the beneficial microclimate of the olive groves to levels similar to those of an arable area. Olive groves are the least effective forest type for reducing land surface temperature, according to the research, but they are more effective than farmland, especially in summer, when air temperatures are at their highest. The same author [163] also investigated the influence of alterations in olive urban forests caused by Xf on ecosystem services, particularly on microclimate and thermal comfort. The analysis showed direct impacts on ecosystem services primarily related to regulatory functions and cultural aspects, since a critical loss of the cultural significance of monumental olive groves also occurred. Additionally, the loss of trees has a detrimental effect on photosynthesis, resulting in decreased oxygen generation and overall CO_2_ absorption [164]. A further amount of CO_2_ is released into the atmosphere as a result of the decomposition of the woody mass and this release occurs much more quickly in fires, to which dry olive groves are particularly vulnerable.

Moreover, olive tree extinction decreases the ecosystem’s capacity to filter the air. Pollination regulation is also impacted by the environmental changes caused by Xf. The pollination of Mediterranean shrubs and other species is affected by their diminished presence after the loss of vegetation. The loss of olive trees and other plant species indirectly affects pollination because the flowers of these plants and other plants are significant food sources for the pollinating insect community, even though the flowers of olive trees do not need insect pollination because many varieties are self-fertile and wind-pollinated. Petrosillo et al. [165] specifically examined at the regional scale how landscape heterogeneity can affect pollination services, using two landscape indicators useful to simultaneously quantify the multiscale landscape composition and configuration. They also evaluated the Xf impact on the pollination services. The multitemporal analysis demonstrated the clear change in the landscape functioning in the provinces affected by the Xf infection (Lecce, Brindisi and Taranto provinces) from 2013 to 2021, highlighting how the loss of permanent land-covers (olive groves) completely altered the stability of the landscape.

Additionally, since 2013, the Xf epidemic also resulted in regulatory requirements and trade restrictions for the Apulia ornamental business. Using cost–benefit analysis (CBA) and life cycle assessment (LCA), Frem et al. [166] evaluated the environmental effects and economic viability of these epidemic-related options in comparison to the conventional production options (CMs) among eight ornamental species. The findings showed that innovative and sustainable ornamental production had a relatively small negative impact on agricultural land use, climate change, fossil fuel and water depletion at the nursery level. It also moderately increased local nursery business profitability in a sustainable economic manner. According to this study, nursery growers could adopt sustainable ornamental production strategies, advantageous to obtain local ornamental and landscape plants with high sanitary quality. Nursery growers are under specific pressure to do so since quarantine pests like Xf are a potential threat. These aspects are also related to the possible promotion of a few underutilized fruit tree varieties with strong commercial potential as control and prevention strategies for the spread of Xfp. These would involve replacing the Xfp-infected olive-growing regions with economically viable fruit tree species. Alhajj Ali et al. [167] evaluated the suitability of almond (*Prunus dulcis* B.), fig (*Ficus carica* L.), hazelnut (*Corylus avellana* L.), kiwifruit (*Actinidia chinensis* P.), pistachio (*Pistacia vera* L.) and pomegranate (*Punica granatum* L.) as fruit tree species immune/resistant to Xfp to be planted within the Xfp-infected olive-growing areas in the Apulia region for possible economic and environmental loss compensation. The investigation revealed that the Xfp-infected olive-growing regions are suitable for most of the suggested fruit tree crops, with varying levels of suitability, according to the specific pedoclimatic conditions. Pomegranate (268,886 hectares), fig (103,975 ha) and almond (70,537 ha) are three fruits that can be grown well in large olive-growing areas, followed by kiwifruit (43,018 ha) and pistachio (40,583 ha). Nevertheless, all possible environmental changes related to the regeneration of Xf-affected areas require specific studies since the different social groups do not all perceive the planned solutions in the same way [168].

Frem et al.’s research [169] analyzed local residents’ preferences for various improvements to olive landscapes and to further research related to Xf. The cultural legacy and aesthetic qualities were found to be the most valued olive landscape services, according to a sociological field survey that included 683 interviewees from three major cities in the Apulia region: Foggia, Bari and Lecce provinces. The results showed that although supportive about restoring the damaged olive groves, citizens were not prepared to spend more for research. Interestingly, Tipaldo et al.’s [170] work investigated the online narrative around the Xf outbreak in Apulia region, as represented by a selection of user-generated content obtained from Facebook, YouTube and Reddit during a time period of 6 years (>16k comments). According to the online Italian users’ discussion analysis, Xf outbreak-related contents resulted to be sharply divided and centered around two conflicts: «expertise vs. politics» and «scientific vs. alternative» solutions.

Table 6 provides a summary of the key findings that show how the Xf pandemic has impacted society, the economy and human well-being.

### 2.7. Olive Germplasm Susceptibility to Xf

The current lack of treatments for eradicating Xf from infected plants together with the above-described strong impacts on the environment, man and society necessitate more concrete containment measures, such as the quest for sources of resistance in olive trees. Therefore, the studies related to olive germplasm susceptibility to Xf also have been considered to complete this review. In California and Brazil, the identification of Xf -resistant germplasm is part of the management strategy of using resistant cultivars and appropriate hygienic-cultural approaches. The possibility of pursuing a similar strategy in the Apulia region emerged following the study by Giampetruzzi et al. [106], who demonstrated that the infected Leccino cultivar had a significantly lower bacterial concentration than that found in Ogliarola salentina and differential gene expression in the presence of infection. Ogliarola cultivar, on the other hand, had much greater bacterial populations and, if infected, had an impressive change in the gene expression due to the water stress situation imposed by Xfp. Luvisi et al. [27] found Ogliarola and Cellina di Nardò to be very susceptible to Xfp. Several mechanisms have been identified that may contribute to this resistance; the vulnerability of olive cultivars to infection is considered to be related to the genetic, biochemical and biophysical properties of the cultivar, as well as the morphology and physiology of the xylem. The bacterium reproduces in plant xylem vessels, disrupting the water transport system and causing the fast decrease that lends the disease its name. De Benedictis et al. [105] suggested that tolerance to Xfp infection is dependent on olive’s ability to control the xylem lumen by blocking it with the tylosis process, as high percentages of occluded vessels were found in these field-grown olives of Ogliarola Salentina, ranging from 16% to 53%. According to Sabella et al. [107], since the initial observations in the field, the Leccino cultivar has demonstrated clear resistance to Xfp, as evidenced by a slower course of the disease compared to other varieties. The analysis of phenolic compounds in healthy and infected leaves of Leccino and Cellina di Nardò revealed a decrease in hydroxytyrosol glucoside and, only in Leccino, an increase in quinic acid, a precursor of lignin that plays a significant role. Further evidence of resistance in Leccino cv came from the studies of Sabella et al. [103], which showed that, compared with the susceptible cultivar Cellina di Nardò, Leccino has a lower susceptibility to cavitation (formation of air bubbles in the xylem) due to a better ability to counteract the decline of hydraulic conductivity imposed by the bacterium. Other investigations, in addition to the presence of lignin associated with Leccino resistance [107], have found changes in the cultivar’s genetics as well as the ionomic and biochemical properties of the xylem, which may contribute to its resistance [171]. In particular, D’Attoma et al. [171] determined the ionic profile of symptomatic and asymptomatic leaves on two olive cultivars (Ogliarola salentina and Leccino), which respond differently to Xf infection. The study found that Leccino cv contained higher manganese (Mn) levels compared with Ogliarola salentina, and these levels were higher in both infected asymptomatic and infected symptomatic leaves. They also showed differences in the ionome, particularly a higher concentration of calcium (Ca) and Mn levels in the Leccino cv and sodium (Na) in both varieties. The susceptible Cellina di Nardò cv experienced significant dysbiosis due to Xfp infection, while the resistant Leccino cv had a more diverse microbial community [111]. The tendency of the endophytic microbiome to succumb to the occupation of the entire ecological niche by Xfp as the infection progresses was also confirmed by subsequent studies, which found that this tendency was more evident in the susceptible Kalamata cv than in the resistant FS-17^®^ [172]. Pavan et al. [122] demonstrated how, after conducting exploratory investigations in olive groves severely afflicted by the bacterium, they located individual plants with a reassuring symptomatic condition. Genetic–molecular analysis has revealed that several kinds already existent in the Salento area, as well as others found throughout the Mediterranean, have resistant properties. It has also been hypothesized that the xylem structure and physiology influence disease resistance. Petit et al. [173] found that the diameter of the xylem vessels probably influences the resilience of the Leccino cv.

Leccino vessels have smaller diameters than the Cellina di Nardò cv. These authors suggested that narrower vessels limit the spread of the pathogen in the plant, due to a slower flow rate, and that their decreased susceptibility to air embolism leads to less cavitation, which is less beneficial to the bacteria’s aerobic metabolism. Surano et al. [174] investigated the physiological responses of Xfp-infected olive trees in susceptible and resistant varieties. Both resistant olive cvs showed less water stress during Xfp infections than the susceptible one, implying that stomatal conductance and stem water potential measures could be used to discriminate between olive genotypes for resistance to Xfp.

Microscopy was used to study the distribution of vascular occlusions in susceptible Cellina di Nardò and resistant Leccino olive cultivars after Xfp infection. Electron microscopy research [125] revealed that Xf subspecies *pauca* strain “De Donno” ST53 spreads via the pit membranes (PMs) of the susceptible cultivar Cellina di Nardò. The study by Savoia et al. [131], which investigated the differential response to Xfp infection in a collection of 100 local Apulian olive genotypes, allowed for the identification of nine putatively resistant genotypes. This evidence represented the first panel of olive germplasm resources and the dissection of the mechanisms involved in plant responses to Xfp infection. However, the processes behind resistance remain poorly understood. X-ray computed tomography is one option for filling some of these knowledge gaps. Walker et al. [175] tested this theory by scanning the stems of four olive cultivars using a technology that segmented vessels to allow for diameter measurements. The findings indicate that susceptible cultivars, which have a higher proportion of larger vessels, are more vulnerable to air embolisms and that, under specific pressure levels, susceptible cultivars’ functional vasculature may be subjected to more stress than resistant cultivars.

In conclusion, focusing on olive germplasm susceptibility to Xf, some general considerations can be drawn for the possibility of rescuing trees from the disease.

Overall, the most important preventative technique is to avoid introducing a bacterium into production. When the bacterium is already present in a given area, disease management requires an integrated approach that may limit its spread by using resistant or tolerant plants, containing the inoculum, treating the possible vector and implementing appropriate cultural practices. The Apulia region (Measure 5.2 of the Italian Rural Development Programme) is funding the replanting of Xfp-tolerant olive tree cultivars, including Leccino and FS-17. “Coratina”, “Pendolino”, “Bella di Cerignola” and “Cipressino” are also noteworthy since, despite the presence of the olive killer bacterium, they do not exhibit desiccation symptoms to date.

Table 7 provides a summary of the main findings in relation to the studies on olive germplasm susceptibility to *Xylella fastidiosa* subsp. *pauca*.

### 2.8. Research Strategies Analysis

**Figure 2:**
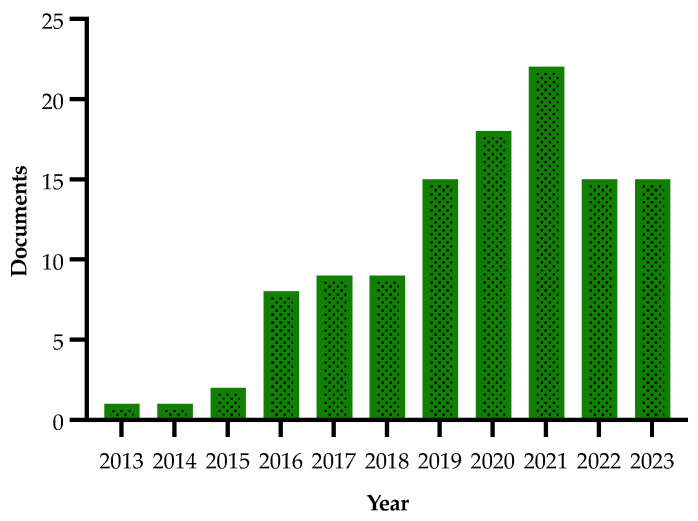
Documents by publication year. Reporting 115 articles.

The presence of *Xylella fastidiosa* in some olive trees in the province of Lecce, Apulia region, was initially reported in October 2013. This is the first outbreak of *X. fastidiosa* in a field setting documented in the European Union. Since then, various topics have emerged, including the pathogen’s biology and genetics, interactions with vectors and host plants, diagnosis and early detection, modeling of the dynamics of infection spread, risk assessment, plant disease management strategies and the socioeconomic impact analysis. The scientific literature, selected using the inclusion and exclusion criteria outlined in this work and focused on research articles, yielded 115 scientific papers over the course of ten years (Figure 2), which were divided into various categories relevant to the topic under investigation. The Xf outbreak has also been covered by a relevant number of reviews in indexed journals, which have been previously listed [16,17,18,19,20,21,22,23,24,25,26,27,28,29,30] in this work but have been excluded from the research strategies analysis.

Seven “research strategies” were selected for the classification, focusing on the following: (a) the biology, genetics and genomics of *Xylella fastidiosa* (*Bacterium Xf*); (b) Xf insect vectors (*Xf vectors*); (c) disease dynamics, modeling and pest risk assessment (*Geographical methods*); (d) disease diagnosis and early detection (*Diagnostic methods*); (e) disease control treatments (*Treatments*); (f) man–environment–society impacts (*Impacts*); and (g) olive germplasm susceptibility to *Xylella fastidiosa* subsp. *pauca* (*Olive susceptibility to Xf*).

**Figure 3:**
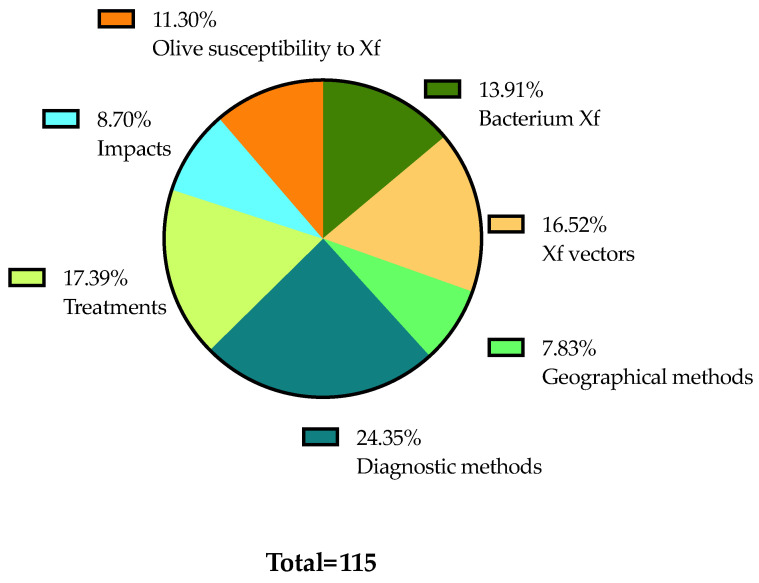
Documents by research strategy subtopics. Reporting 16 articles related to *Bacterium Xf* subtopic, 19 to *Xf vectors* subtopic, 9 articles related to *Geographical methods*, 28 articles for *Diagnostic methods*, 20 articles related to *Treatments* subtopic, 10 for *Impacts* on environment and society and 13 articles related to *Olive germplasm susceptibility to Xf* subtopic.

Figure 3, which depicts the scientific articles divided by research strategy, shows that in the last decade scientific research has primarily focused on the *Diagnostic methods,* including the application of possible techniques for early disease diagnosis. These include bacteriological isolations from fresh symptomatic tissues, biological assays and biochemical methods, serological methods, electron microscopy and molecular biology, with 28 documents accounting for 24.35% of all the literature. The further focus in the research subject concerns *Xf vectors* investigations (16.52%); while all articles on the *Treatments* include fewer than 1/5 of the overall considered scientific production (17.39%). Characterization of the disease responsible microorganism *Bacterium Xf* is found in 13.91% of the documents, while documents related to studies on *Olive germplasm susceptibility to Xylella fastidiosa* subsp. *pauca* represent 11.30% and the remaining papers concerning *Geographical methods* and *Impacts* account for 7.83% and 8.70% of the covered literature, respectively. It should be also considered that our classification arrangement, according to the “research strategies” in this review, generally refers to a single allocation of the examined papers in a specific single “strategy”. Nevertheless, there are some cases of overlapping “research strategies” in the same published scientific work. This concerns a minimum number of articles and is essentially limited to *Diagnostic methods* and *Olive germplasm susceptibility to Xf*, sharing eight papers among those here reviewed [27,103,105,106,107,122,125,129]. Therefore, the number of items indicating the overall examined studies defined in the different “research strategies” (n. 107) is simply increased by eight units compared to the overall number of reviewed papers (n. 115).

**Figure 4:**
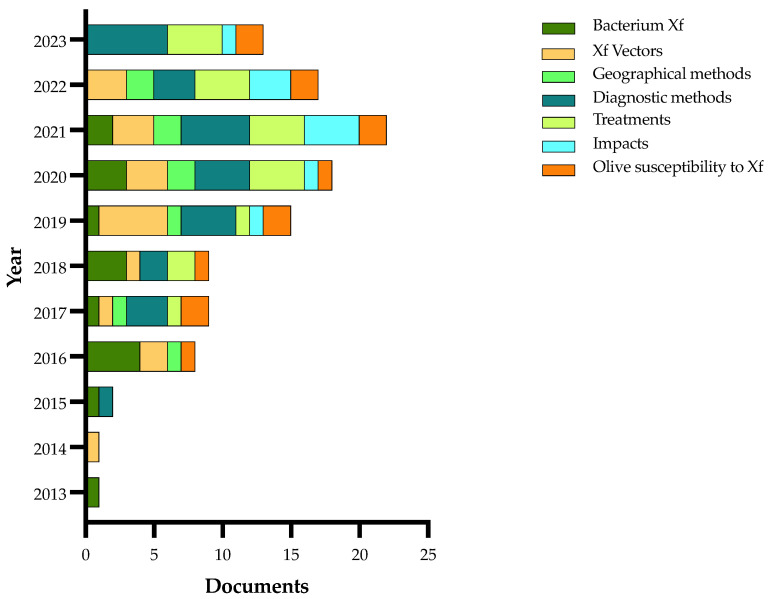
Documents by year in relation to research strategy subtopics.

Figure 4 illustrates how the number of articles on the subject of the review has increased over time, also showing the specific disaggregation according to the identified research strategies. The scientific investigations, initially focusing on knowledge of the bacterium and vectors (*Bacterium Xf* and *Xf vectors*), from the beginning of 2015 moved on to studies on advanced technologies for early detection and diagnosis, including image analysis techniques (*Diagnostic methods*). As seen by the expanding number of publications over the years, the lines of research have concentrated primarily on the quest for disease diagnostic and/or phytopathogen identification methods (*Diagnostic methods* and *Bacterium Xf*). Since 2015, studies concerning the use of resistant cultivars have also been conducted (*Olive susceptibility to Xf*). The first results on the specific control strategies employed to contain the further spread of the disease (*Treatments*) were published only in 2017, soon after the start in 2016 of the first approach to *Geographical methods* investigations and before the first *Impacts*-related research (2019). Interestingly, after 2017, the *Treatments*-related papers publication continued yearly until 2023, but in considerably fewer numbers compared to investigations including the other combined research strategies, in particular *Diagnostic methods*, *Bacterium Xf* and *Xf vectors*.

Examining the temporal profiling of the different research strategies identified here, according to their appearance in the published literature, the work related to microorganism characterization (*Bacterium Xf*) occurs first.

Studies on the genetic characteristics of the bacterial population linked with epidemics or outbreaks of infection are critical to understanding disease epidemiology, including phytopathologies like Xf. Indeed, genetic knowledge has aided in the identification of the factors that characterize the pathogen’s spread, as well as the acquisition of data and information on adaptation to specific climatic conditions, space–time evolution, the identification of potential routes of introduction and the biology of the various strains.

**Figure 5:**
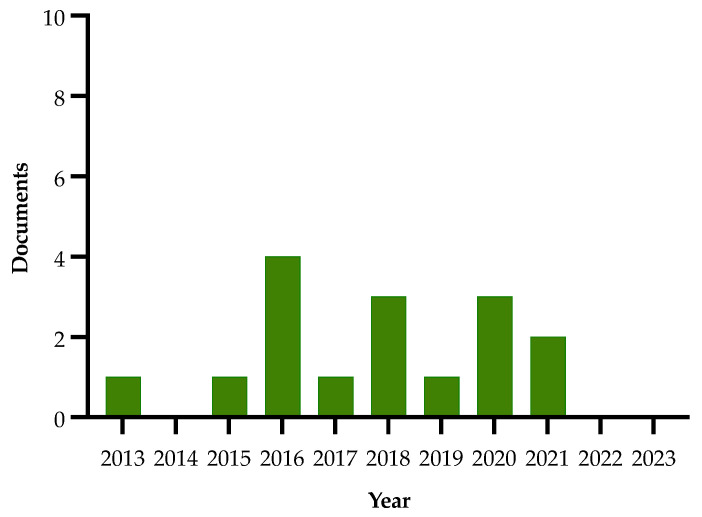
Documents per year involving the bacterium *Xylella fastidiosa* (*Bacterium Xf*). Reporting 16 articles.

On this topic, scientific works (overall 16 documents) have expanded throughout time, allowing us to greatly improve our understanding of Xf’s genetic diversity on a worldwide scale, as well as to contribute to the advancement of tools for exploring this diversity (Figure 5). The bacterium characterization published work, which started in 2013, [32] almost steadily continued in the following years reaching a maximum in 2016 when phylogenetic analyses were also conducted [44,46,47,48].

By continuing the temporal profiling of research strategies, the relevance and role of vectors in the spread of the bacterium was discovered (*Xf vectors*). Xf needs an insect vector to move the bacteria from an infected plant to a healthy one.

**Figure 6:**
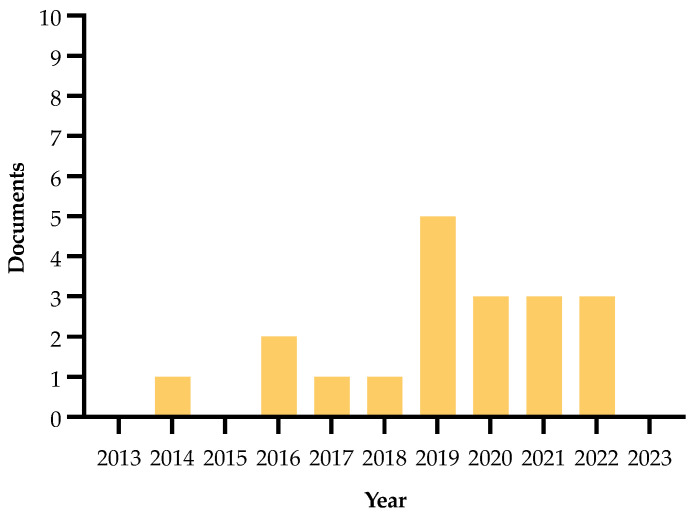
Documents per year involving the vector (Xf vectors). Reporting 19 articles.

Three species of spittlebugs (Family Aphrophoridae) have been discovered as vectors in Apulian olive trees, the most common of which is the medium spittlebug (*Philaenus spumarius*). The relevance and role of vectors in the spread of the bacterium depends not only on the ability and efficiency to transmit the pathogen, but also on the ecological and ethological characteristics, the range of preferential hosts, the fluctuation and dynamics of the population on cultivated and weedy plant species, interactions with other epidemiological components of the disease, pathogenetic dynamics in the hosts, the Xf genotype and environmental conditions. The research in the literature (overall 19 documents) aimed at collecting data on the possible risk posed by these vector species in the event of Xf introduction and dissemination. The first papers about vector insects transporting the Xf bacteria in the plant’s xylem date back to 2014 [7]. Over time, research has allowed us to look deeper into the progression of the biological cycle as well as our understanding of host plants. The findings from this study have shown the control of vectors to be part of the plan to limit the spread of the bacterium (Figure 6). In 2019, scientific production relating to the survey of the various stages of development of the biological cycle of Xf vector insects increased, reaching a peak. The studies provided useful indications on the timing of implementation of interventions for monitoring, but most importantly for juvenile and adult control [70,71,72,75,77]. Thereafter, there has been a minor (compared to 2019) but steady production of vector-related studies.

**Figure 7:**
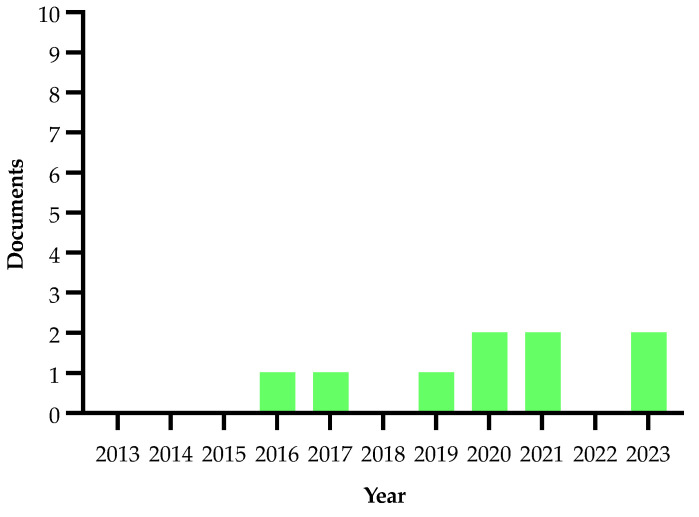
Documents per year based on geographical methods. Reporting 9 articles.

Understanding the spatiotemporal dynamics of infections and sickness associated with Xf infections in olive groves is also critical for developing an effective management strategy (*Geographical methods*). The first insights into the space–time spread of Xf infections were obtained in the Apulian area through field surveys conducted over time beginning in 2016 [92], employing various and effective risk assessment and prevention methods and instruments (Figure 7). The published papers on *Geographical methods* continued in the following years until 2023, reaching a maximum from 2020 to 2021 [94,95,96,97]. The use of simulation models found in scientific works (overall nine documents) that provide ecological and epidemiological models resulted in more accurate predictions of the bacterium’s potential for establishment and diffusion, providing key elements for more targeted interventions (monitoring strategies, priority of areas/hosts for investigations and sampling, eradication, criteria for area delimitation, etc.).

As previously mentioned, the most relevant number of publications (n = 28) published in the whole decade examined (2013–2023) are about diagnostic protocols and early diagnosis (*Diagnostic methods*), since various research groups have developed diagnostic processes for very sensitive detection of Xf in host plants and vectors.

**Figure 8:**
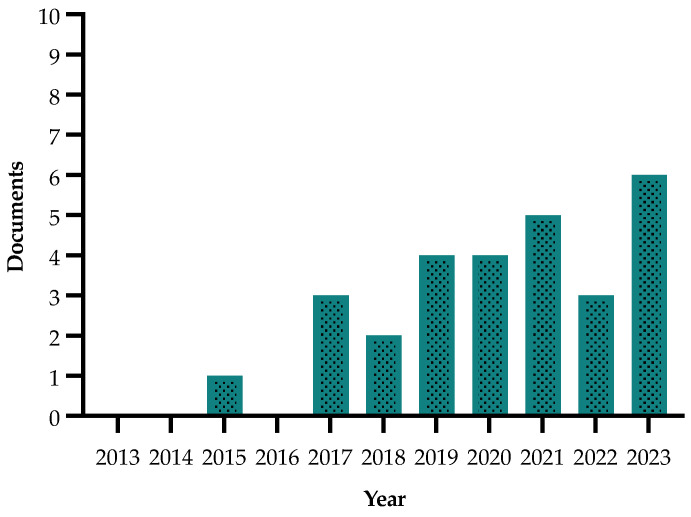
Documents per year based on the use of diagnostic systems. Reporting 28 articles.

In the overall context of diagnosis, early detection of Xf infections (i.e., before the appearance of distinct and obvious symptoms) is one of the research’s key and ambitious goals, with the first publication in 2015 [102] and which has been consistent since 2017 [27,105,107,108,109,110,111,113,114,115,116,118,119,121,122,123,124,125] (Figure 8). Published documents relating to the various diagnostic techniques used for Xf detection have seen a nearly constant increase, reaching a maximum in 2023 [127,128,129,130,131,132].

**Figure 9:**
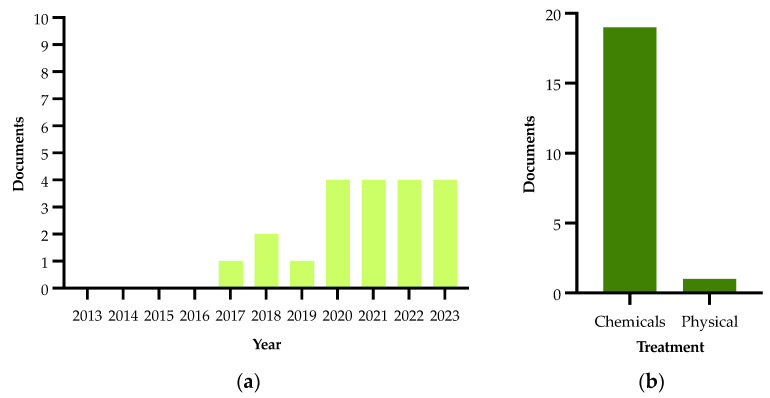
(**a**) Documents per year based on treatments against infected plants. Reporting 20 articles. (**b**) Total documents (n = 20) related to research subtopic “*Treatments*” describing the application of chemical (n = 19) and physical (n = 1) treatments.

The above-described diagnosis strategy studies, which are well reported in the scientific literature, do not seem to be paralleled by investigations on the possible microorganism’s containment actions (*Treatments*). The latter could have been profitably considered along with the drastic compulsory tree eradication procedures dictated by the epidemic-related regulations [176,177,178]. The series of scientific documents relating to the *Treatments* (overall 20 documents) (Figure 9a) began in 2017 [117] and stayed consistent from 2020 until 2023. Figure 9b shows that 19 out of 20 documents involve studies on various chemical formulations/molecules and biocontrol agents (Chemicals) and only one involves mechanical operations for Xf control (Physical) [152].

**Figure 10:**
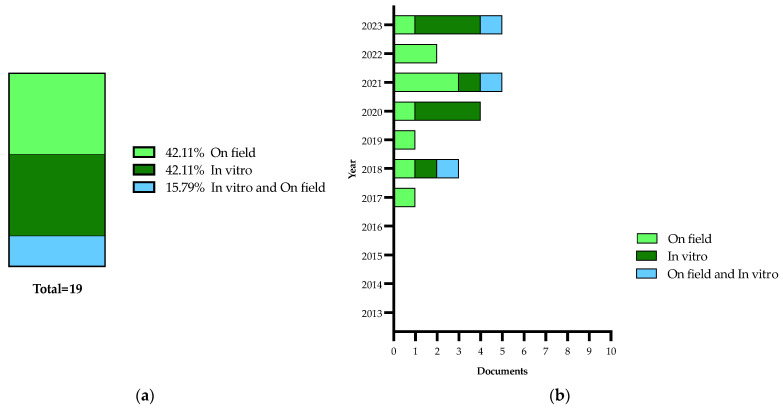
(**a**) Documents describing the application of chemical treatments (n = 19), with the application of on field, in vitro and both techniques; (**b**) documents by year in relation to the application site of chemical treatments (on field, in vitro, on field and in vitro).

The experiments conducted and currently in progress for the research of therapeutic solutions and integrated control strategies aimed at reducing the impact of infections have involved experiences both in vitro and on field, aimed at striking down the bacterium in plants, using chemical–mineral formulations and compounds, natural products derived from plants and microbial antagonists. In particular, Figure 10a depicts the subdivision of treatments by the field of intervention, highlighting how field assessments (on field) slightly prevail as the favored approach for the control of Xf (42.11%) with 8 documents, out of 19 total. Among the various on field treatments, which include the use of mineral solutions [153], as well as vegetable oils and extracts from various botanical species [145], zinc and copper administration as the citrate biocomplex prevails [117,135,136,139,143,144,147,149,151]. In addition, 42.11% of scientific works (eight documents) refer to in vitro testing with the aim of selecting potential antimicrobial compounds to be used in infected plants [137,142,148,150,154] or identifying antagonistic organisms potentially deployable as biocontrol agents against Xf [140,141]. Both fields of intervention (on field and in vitro) are jointly reported in three documents (15.79%), where the research included field testing after in vitro effectiveness testing of the same compounds [135,143,152].

Figure 10b illustrates how the number of articles on the treatment subtopic has increased over time; the studies, initially focusing on the application of on field techniques, from 2017 moved to studies that also use in vitro techniques and both fields of intervention (on field and in vitro). In particular, the number of published documents related to on field testing results in an average constant increase, reaching a maximum in 2021 [143,144,145,146]. Furthermore, documents that indicate the application of in vitro techniques reached the maximum level in 2023 [150,153,154].

**Figure 11:**
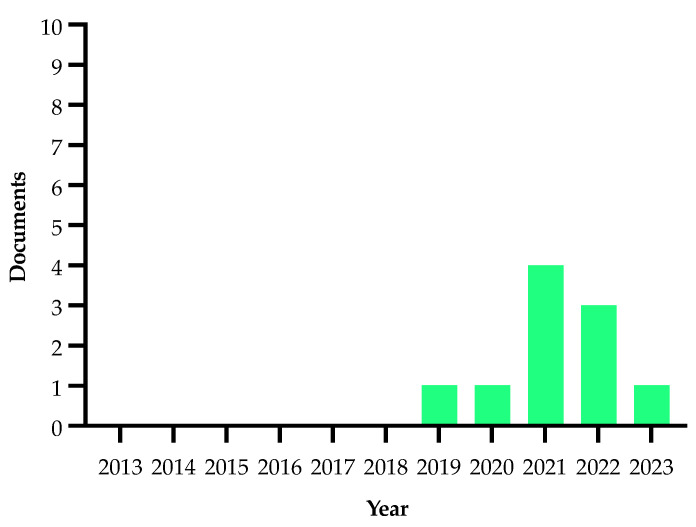
Documents per year on impact on the environment and society. Reporting 10 articles.

The temporal profiling of research strategies has been continued by examining the impacts that Xf has had on humans and the environment (*Impacts*). Effectively, the expansion of Xf has a wide-ranging influence on agricultural productivity and the landscape, resulting in enormous economic losses for society, humans and the environment. On this topic, scientific documents (overall 10 documents) have expanded throughout time, trying to describe the psychological and sociodemographic aspects related to the beliefs and intentions of olive growers’ about Xf control in the Apulia region (Figure 11). The analyses of *Impacts*-related published work, which started quite recently in 2019 [163], continued in the following 4 years, reaching a maximum in 2021, when farmers’ perceptions of the Xf pandemic were studied [166].

**Figure 12:**
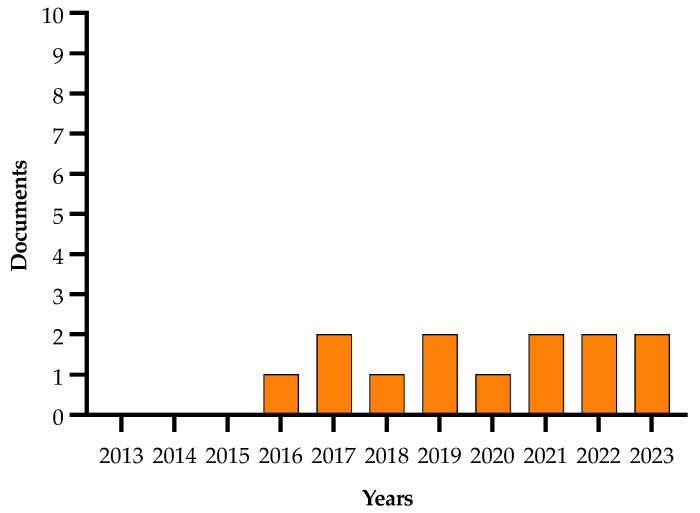
Documents per year on olive germoplasm susceptibility to Xfp. Reporting 13 articles.

The last considered research strategy subtopic (*Olive susceptibility to Xf*) focuses on the identification of cultivars showing tolerance/resistance to the pathogen. On this topic, 13 scientific documents have been published. These were published starting from 2016 with the first attempt to characterize the transcriptome profiling of two olive cultivars Ogliarola salentina and Leccino, behaving differently in response to infection by Xfp [106] (Figure 12). The published papers on this subtopic continued with a limited, but constant, production in the following years until 2023, indicating a continuous interest for this specific research field. The latter is still a major focus point for ongoing studies, considering that, at the present, only two cultivars are officially indicated as suitable for olive orchard replanting in the infected areas.

In conclusion, the research strategy analysis performed on the literature included in this review reveals that the majority of scientific efforts have been directed toward fields related to diagnosing rather than treating plant illness. In fact, 53 of the 115 works included in the examined literature selected with the qualitative synthesis refer to direct or indirect diagnosis related subtopics. These include *Diagnostic methods,* leading the group with 28 papers, followed by the studies involving the *Bacterium Xf* (16 articles) and those on *Geographical methods* applied to the disease investigations (9 articles). Vector identification and studies constitutes another relatively well-represented scientific field (*Vectors Xf*), with 19 out of the 115 investigations focused on the specific topic. Only 10 research studies focused on the environmental, human and societal impacts and 13 related to study olive germplasm susceptibility to Xfp.

**Figure 13:**
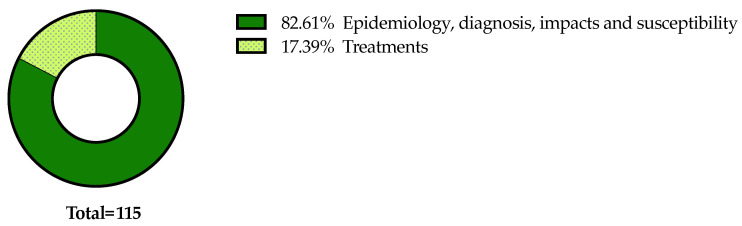
Scientific literature report between 2013 and 2023—n. 95 articles on epidemiology and diagnosis (bacterium, vector, geographical distribution, impact, diagnostic methods and olive germoplasm susceptibility to Xf) focus and n. 20 articles for treatment against infected plants.

It is important to note that only 20 (17.39%) of the 115 papers of the examined literature, in the specific qualitative review, report on treatments for the bacterium (Figure 13). This result is even more striking considering the comparison with the comprehensive literature produced on the topic further subject to the qualitative selection in this review. Overall, the 20 papers concerning the use of treatments intended to apply therapeutic procedures against infected plants constitute only 6.36% of all of the 314 papers published between 2013 and 2023 on the topic of *Xylella fastidiosa* in Apulia. On the other hand, limiting the comparison to the papers of the qualitative selection is very interesting considering that only 12 out of the above-described 20 are papers directly related to or include on field treatments. Moreover, it should also be considered that, excluding the physical treatments (1), only 11 studies (12%) directly refer to on field treatments involving the use of specific substances suggested for possible phytotherapy. Among the latter, two refer to the use of natural substance derivatives and the others to a Zn and Cu citric acid biocomplex.

## 3. Methods

### Data Collection and Data Elaboration

A search for articles published up to 31 December 2023 was conducted to assess the literature covering all facets of the Xf outbreak in Apulia from 2013. The evaluation was conducted in accordance with the primary components listed in the PRISMA checklist 2009. We searched on Scopus and Web of Science (WOS) for articles published from January 2013 to December 2023, by using the following keywords “*Xylella*” AND “Olive” OR “Olea” AND “Apulia” OR “Puglia” in different combinations. Separately, in each database, the studies were chosen for the bibliographic search based on their titles or abstracts and then the whole articles were examined. First, duplicates between databases were removed. The results of the separate research were loaded into the Rayyan screening tool (http://www.rayyan.ai (accessed on 3 January 2024)) for further analysis deleting duplicates between databases. Subsequently, the records were screened by article type including only original full-text papers in the English language with the exclusion of the reviews, both systematic and narrative, as well as metanalyses, conference and proceedings papers, notes, book chapters, short surveys and editorial material.

Following that, articles in which Xf was not associated with Apulian olive trees, studies in which Xf was not the main topic, articles conducted in geographic areas other than the Apulia region, articles of a political/governmental nature, letters, perspective and short communication, were excluded. Furthermore, European Food Safety Authority (EFSA) statements were excluded. For each examined work, we studied the original paper, and we extrapolated the following information:

- Authors;
- Publication year;
- Country of publication;
- Research strategy;
- Key conclusion.

In particular, “research strategies” were identified according to the following subtopics:

- Studies involving genetic and genomic features of *Xylella fastidiosa* subsp. *Pauca*;
- Studies involving the biology and spread of the vector *P. Spumarius*;
- Studies based on geographical and epidemiological methods;
- Studies based on the use of diagnostic techniques;
- Studies based on in vitro assays and treatments against infected plants;
- Studies on *Xylella fastidiosa* subsp. *Pauca* impact on the environment and society;
- Studies on olive germplasm susceptibility to *Xylella fastidiosa* subsp. *pauca*.

## 4. Conclusions

In 2013, one of the most virulent *Xylella* strains ever identified was introduced into the Apulia region. Farmers, phytosanitary officials and the research community were confronted with a massive problem that required immediate recognition of its gravity and complexity, as well as timely intervention. The bacterium has caused irreparable damage to the territory over the last ten years. The time it will take to rebuild the territory after such a disaster is undoubtedly long, but in the meantime, the research and dedication of various olive growers is laying the groundwork for the olive sector’s regeneration and beyond. This review of the scientific literature emphasizes the difficulty of finding effective methods for the treatment of the infected olive trees, which have been very few in recent years when compared to the attention paid to diagnosis methods. The prevalence of the latter could be closely linked to the need to implement and apply phytosanitary measures, imposed by the phytosanitary authorities, in a timely manner. On the other hand, this review clearly indicates the minor role played by the treatment-dedicated research strategies. Nevertheless, the results of the limited research on effective treatments on olive trees also report positive indications on the possibility to contain the disease. This suggests the need to point the scientific efforts towards an increase in the research for possible treatments against the bacterium in the present case and when generally dealing with similar situations. Indeed, wider treatment-focused research and a timely consideration of the possible positive results are key steps in fighting the disease. These basic commitments also require adaptive management of the legislation related to the novel specific epidemic situation, which could limit the eradication of olive trees and all host plants surrounding diseased trees. Many centenary and monumental olive trees, as well as the splendid scenery to which they contribute, could thus be saved. Fighting the spread of Xfp and protecting Apulia’s olive producing and landscape heritage will require relentless dedication and an integrated strategy. The sustainable regeneration of the ecosystem through models that promote biodiversity will play an important role in the future in the reconstitution of the balance between man and the environment. It requires a multidisciplinary approach to understand how environmental changes can impact human health and to implement containment and primary prevention actions against such effects, which can be transferred to public health programs and policies. From the perspective of One Health, it is necessary to abandon the usual anthropocentric vision. Instead, it is important to increase awareness that the well-being of man is closely connected to the health of ecosystems; all its inhabitants must have equal valor if a sustainable resilient and durable environment is to be created. In conclusion, the aim of this paper was to create a thorough methodical analysis of the literature reports related to the use of different research strategies focused on the Xfp outbreak in Apulia from 2013 to the present. In particular, this review highlighted possible unbalanced efforts that occurred when performing different research strategies. Among all the considered approaches (attempts to identify the bacterium; studies to recognize and control the vector; geographical methods; diagnostic techniques; therapeutic procedures; impacts on the environment, man and society and olive germplasm susceptibility investigation) here, an increase in research for possible treatments against the bacterium also focused at keeping plant productivity is strongly suggested. In this respect, the reported review of the scientific literature results here could provide further useful information for the development of improved surveillance programs and control strategies, in addition to early detection. Among the potential future directions, it is therefore necessary to continue exploring new strategies for the management of Xfp, including genetic studies for resistance to Xfp and control methods for vectors, but, above all, an enhancement of treatment-focused investigations, also aimed at maintaining plant productivity, appears pivotal.

## Figures and Tables

**Table 1:** Studies involving the identification and characterization of Xylella *fastidiosa* (*Bacterium Xf*).

Authors and Year	Method	Main Findings
Carlucci et al., 2013 [32]	Definition of phytosanitary emergency	Xf in olive plants in the Mediterranean area
Giampetruzzi et al., 2015 [43]	Genomic DNA investigation	They determined the draft genome sequence of the Xf CoDiRO strain; it is associated with olive quick decline syndrome (OQDS) and characterized by extensive scorching and desiccation of leaves and twigs
Mang et al., 2016 [44]	PCR assays	Nucleotide variation present on gyrB gene allowed separation of Xf subsp. *pauca* from the other subspecies multiplex and *fastidiosa*. The Xf strain from Apulia region was included in the subspecies *pauca* based on three gene phylogenetic analyses
Loconsole et al., 2016 [46]	MLST and phylogenetic analyses	They reported new foci as well as host plant species positive with Xf, including cherry, myrtle leaf and rosemary; all were found to be infected with the same sequence type of this bacterium (ST53 or CoDiRO strain)
Marcelletti and Scortichini, 2016 [48]	Genome-wide approach	This study strongly supports the possibility of the introduction of Xf into southern Italy through coffee plants grown in Central America
Martelli et al., 2016 [47]	Definition of phytosanitary emergency	The bacterium was isolated in culture and identified as a genotype of Xf subsp. *pauca*, molecularly identical to an isolate from Costa Rica. *Philaenus spumarius* (meadow spittlebug), a froghopper quite common in the Salento area where it thrives on olives, was identified as the main vector
Saponari et al., 2017 [51]	PCR assays	Needle-inoculation experiments under different environmental conditions proved that the Salentinian isolate De Donno belonging to the subspecies *pauca* is able to multiply and systemically invade artificially inoculated hosts, reproducing symptoms observed in the field
Cella et al., 2018 [49]	Phylogenetic and evolutionary analysis	Xf strains belonging to Xf subsp. *pauca* and subsp. *sandyi* were reported to infect olive trees and coffee plants, respectively. The phylogeographic analysis also revealed and confirmed these two different ways of provenience
Ramazzotti et al., 2018 [56]	VNTR, RAPD and rep-PCR (ERIC and BOX motifs) analyses	Genome-wide indices ANIm and dDDH indicated that the three isolates of Xf from Salento (Apulia, Italy), namely Salento-1, Salento-2 and De Donno, whose complete genome sequence has recently been released, share a very recent common ancestor
Scala et al., 2018 [57]	LC-TOF and LC-MS/MS techniques	Different lipid compounds present a clear distribution pattern within the infected plant tissues compared to the uninfected ones
Scortichini and Cesari, 2019 [58]	Serological and molecular techniques	The cultivars “Nociara”, “Cima di Melfi” and “Cellina di Nardò” showed the highest occurrence of decline symptoms
D’Attoma et al., 2020 [53]	In vitro behavior of the strain and compare its relevant biological features with those of the strain Temecula1	The study showed that the strain De Donno did not show fringe on the agar plates, produced larger amounts of biofilm and had a more aggregative behavior than the strain Temecula1
Mazzaglia et al., 2020 [52]	Molecular techniques (a multiple locus VNTR analysis assay)	A total of 37 TR loci were amplified on the genomic DNAs of the Apulian strains from representatives of Xf subspecies and directly on DNA extracted from infected plants
Scala et al., 2020 [59]	LC-MS/MS and multiple reaction monitoring (MRM) methods	This study provides novel insights on OQDS lipid hallmarks and on molecules that might modulate biofilm phase in Xf subsp. *pauca*
Firrao et al., 2021 [60]	BLASTP analysis	The results of the pan-genome analyses stressed the additional relevance of environmental DNA in shaping their genomes
Sicard et al., 2021 [61]	Genomic and phylogenetic analysis	They first showed that the outbreak in Apulia is due to a single introduction from Central America that was estimated to have occurred in 2008

**Table 2:** Studies involving the characterization and control of *Xylella fastidiosa* subsp. *pauca* vector.

Authors and Year	Method	Insects Tested	Main Findings
Saponari et al., 2014 [7]	PCR assays	Ps and El	Ps identified as a vector of Xf infecting olive trees in the Salento Peninsula
Moussa et al., 2016 [69]	PCR assays	Ps, Nc and El	Ps was the dominant species with the highest adult abundance in summer months
Cornara et al., 2016 [8]	Transmission tests and PCR assays	Ps	The number of PCR-positive Ps on each plant was positively correlated with the plant infection status. These data show that field-collected Ps have high rates of Xf infection and are competent vectors
Cornara et al., 2017 [9]	Real-time PCR	Ps and Nc	Data demonstrated that Ps acquired and transmitted Xf from several host plant species in the field, with the highest acquisition rate from olive, polygala and acacia
Cornara et al., 2018 [26]	EPG-assisted characterization of P. spumarius female feeding behavior	Ps	Ps feeding behavior can be described by five main distinct waveforms including pathway, xylem contact/pre-ingestion, xylem sap ingestion, resting, interruption within the xylem phase
Bodino et al., 2019 [70]	Field surveys	Ps, Nc and *Aphrophora alni*	Data on the life cycle of spittlebugs within an olive agroecosystem with Ps adults being abundant on the herbaceous cover and olive trees in late spring, then dispersing to wild woody hosts during the summer and returning to the olive groves in autumn
Fierro et al., 2019 [75]	Lattice model proposal with numerical simulation and field surveys	Ps, Pi and Nc	A lattice model was constructed to simulate the bacterium/vector/tree infection interplay under different control actions, in order to explain to what extent the infection can be mitigated even in un-favorable conditions
Cavalieri et al., 2019 [71]	Quantitative PCR assays	Pi *Drosopolous* and *Remane,* Nc and *Latilica tunetana* (Matsumura) (Issidae)	The vector-mediated transmission experiments conducted over a two-year period showed that, besides Ps, two additional spittlebug species are competent vectors of the strain of Xf subsp. *pauca* ST53
Dongiovanni et al., 2019 [77]	Field surveys (randomized plant sampling and quadrats sampling)	Ps and Nc	The botanic families presenting the highest number of plants infested by Ps nymphs were Asteraceae, Fabaceae and Apiaceae, peaking in early April
Cornara et al., 2019 [74]	Field surveys, quantitative PCR assays and EPG procedures	Ps	First insights into the transmission dynamics of the bacterium Xf by Ps: acquisition occurs at a very low rate during the first minutes when the insect is ingesting the xylem sap
Bodino et al., 2020 [78]	Field surveys	Ps, Nc and *Aphrophora alni* (L.)	Ps was the predominant species in Apulia olive groves, however principal alternative woody hosts are *Quercus* spp. and *Pistacia* spp.
Ganassi et al., 2020 [86]	Field surveys and electroantennographic recording (EAG)	Ps	The electrophysiological and behavioral responses of adult Ps towards some essential oils and related plants were reported
Cornara et al., 2020 [72]	Transmission tests and quantitative PCR assays	*Platypedia minor*, *Cicada orni*	Data suggest that the cicada species have no, or a negligible, role in the natural spread of Xf
Bodino et al., 2021 [79]	Field surveys (MRR experiments)	Ps	The dispersal of Ps is limited to some hundreds of meters throughout the whole year, although it can be influenced to a great extent by the structure of the agroecosystem (olive groves and meadows)
Cornara et al., 2021 [80]	Field surveys	Nc, Ps and Pi *Drosopoulos* et Remane	Reported data on the presence and abundance of spittlebugs during the year in four different habitats interspersed with cultivated orchards within a natural area in Apulia
Bodino et al., 2021 [81]	Quantitative PCR assays	Ps	Ps is a competent Xf vector to olives throughout its adult life; bacterial load in the vector foregut increases during the first 2–3 weeks after acquisition
Lahbib et al., 2022 [66]	Field surveys and light microscope and SEM observations	*Philaenus* species	The study revealed the true phenology of *Philaenus* species individuals, allowing for the classification of ambiguous individuals
Bozzo et al., 2022 [82]	Spatial pattern clustering methodological approach	Ps	Spatial variation and territorial differentiation may differ from zone to zone in the same invaded area
Cascone et al., 2022 [87]	Field surveys and olfactometer bioassay	Ps	The response of Ps towards olive varieties was sex-dependent: males were totally unresponsive whilst females were attracted by Ogliarola, Rotondella and Frantoio

**Table 6:** Studies on *Xylella fastidiosa* subsp. *pauca* impact on the environment and society.

Authors and Year	Methods	Impact	Main Findings
Semeraro et al., 2019 [162]	Methodological analysis using the environmental impact assessment (EIA) and the geographic information system software QGIS (qgis.org, accessed on 20 May 2019)	Impact of changes in olive urban forests affected by Xf on ecosystem services	The study revealed that direct effects on ecosystem services are principally linked with regulation functions and cultural services
Brunetti et al., 2020 [161]	Mathematical model and numerical simulations	Improvement of control strategies within the integrated pest management framework	Implemented mathematical model suggests that a removal of a suitable amount of weed biomass (reservoir of Xf) from olive orchards and surrounding areas was the most efficient strategy to control the spread of OQDS
Semeraro et al., 2021 [163]	Landsat data and moderate resolution imaging spectroradiometer (MODIS) images (https://lpdaac.usgs.gov/dataset_discovery/modis/modis_products_table, accessed on 12 October 2020).	Impact of destruction of olive groves by Xf on local climate change	This study analyzed how the destruction of olive groves by Xf affects local climate change
Frem et al., 2021 [166]	Choice experiment	Impact on the provision of olive landscape services which yield changes in components of public’s well-being	The study revealed that for the local citizens interviewed, the most appreciated olive landscape services are cultural heritage and aesthetic values
Ali et al., 2021 [158]	Environmental risk assessment	Short- and long-term impacts of this disease and the control measures against it on ecosystem services	The study provided the first assessment of the wider environmental impacts of Xf subsp. *pauca*
Tipaldo et al., 2021 [170]	Computer-assisted text analysis	Impact on social media	They find that discourses on Xf are strongly polarized and structured around two conflicts: «expertise vs. politics» on one hand and «scientific vs. alternative» solutions on the other
Petrosillo et al., 2022 [165]	Multiscale spatial and temporal analysis	Planning of landscape functionality recovery	The multitemporal analysis has allowed the authors to show the evident change in the landscape functioning in the provinces interested by the infection of Xf from 2013 to 2021
Frem et al., 2022 [169]	Environmental implications assessment (LCA)	Impact assessment	This research provided a useful decision support, through a study to assess the environmental implications and economic viability of novel and sustainable ornamental production versus the conventional production options
Schneider et al., 2022 [157]	Climatic suitability map, economic model	Impact assessment	They developed a spatially bio-economic model to compute potential future economic impact of the Xfp strain. For Italy, the potential economic impact over 50 y ranges from 1.9 to 5.2 billion Euros for the economic worst-case scenario
Alhajj Ali et al., 2023 [167]	Land suitability analysis and GIS	Practical containment measures against the diffusion of Xfp	Their results can help in the selection of the right immune/resistant tree species for replanting in Xfp-infected zones

**Table 7:** Studies on olive germplasm susceptibility to *Xylella fastidiosa* subsp. *pauca*.

Authors and Year	Methods	Olive Cultivars Tested	Main Findings
Giampetruzzi et al., 2016 [106]	Molecular analysis	Ogliarola salentina and Leccino	Xfp elicits a different transcriptome response in the two cultivars, which determines a lower pathogen concentration in cv. Leccino
De Benedictis et al., 2017 [105]	Genotype identification and microscopy analysis	Ogliarola salentina, Cellina di Nardò and Leccino	Occlusions were caused by tyloses and, as observed in Leccino plants, they are not a marker of tolerance/resistance to the disease
Luvisi et al., 2017 [27]	Molecular analysis	Cellina di Nardò, Ogliarola salentina, Frantoio and Leccino	Differences in the induced responses of phenolic compounds (hydroxytyrosol glucoside and quinic acid) among cultivars suggest that they play defensive roles in olive tree response to Xf infection
Sabella et al., 2018 [107]	Molecular and phenolic analyses	Cellina di Nardò and Leccino	Results suggest a critical role for lignin in *X. fastidiosa* tolerance of cv. Leccino
D’Attoma et al., 2019 [171]	Inductively coupled plasma optical emission spectroscopy (ICP-OES)	Ogliarola salentina and Leccino	Their analyses showed that the ionome differences in the two varieties, particularly a higher concentration of calcium (Ca) and Mn levels in the Leccino cultivar, contribute to protection against disease caused by *X. fastidiosa* infection
Sabella et al., 2019 [103]	SEM-EDX analysis and molecular analysis	Cellina di Nardò and Leccino	Leccino cv was anatomically less susceptible to cavitation and it may also be able to activate more efficient refilling mechanisms, restoring vessel hydraulic conductivity
Giampetruzzi et al., 2020 [172]	Molecular and metagenome shotgun sequencing (WMSS)	Kalamata and FS17	The progression of the infections detected in both cultivars suggested that Xf tends to occupy the whole ecological niche suppressing the diversity of the endophytic microbiome, but this trend was mitigated in the resistant cultivar FS17
Pavan et al., 2021 [122]	Molecular analysis	Nocellara Messinese, Frantoio, Bella di Spagna, Pendolino, Cellina di Nardò, Ogliarola Salentina and Leccino	Their results indicated the possibility to characterize resistance to Xf in cultivars genetically related to putatively resistant plants
Petit et al., 2021 [173]	Characterization and measurements of functional xylem anatomy	Cellina di Nardò and Leccino	The higher air embolism vulnerability of the larger vessels in Cellina di Nardò possibly facilitates Xf infection compared to Leccino
Surano et al., 2022 [174]	Measurements of stomatal conductance and stem water potential	Cellina di Nardò, Leccino and FS17	In this study, both resistant olive cultivars showed lower water stress upon Xfp infections, compared to the susceptible one
Montilon et al., 2022 [125]	Electron microscopy analysis	Ogliarola salentina, Cellina di Nardò and Leccino	Their results suggested that Xfp ST53 exploited the pit membranes of the susceptible cultivar Cellina di Nardò to spread systemically. In Leccino, occluded vessels were mainly filled by callose-like granules that tightly entrapped XfDD cells
Savoia et al., 2023 [131]	Molecular test (qPCR, genotyping)	Leccino and Cellina di Nardò	They identified nine putatively resistant genotypes that represent the first panel of olive germplasm resources
Walker et al., 2023 [175]	Molecular analysis and X-ray computed tomography	Ogliarola, Koroneiki, Leccino and FS17	Their results showed susceptible cultivars, having a greater proportion of larger vessels, are more vulnerable to air embolisms

## Data Availability

Not applicable.
